# Supplementary material for: Small Molecule Enhancers of Endosome-to-Cytosol Import Augment Anti-tumor Immunity
Source: Cell Rep. 2020 Jul 14;32(2):107905. doi: 10.1016/j.celrep.2020.107905 (PMC7370168; doi:10.1016/j.celrep.2020.107905)
Supplement: Document S1. Figures S1–S6 [file mmc1.pdf]

Cell Reports, Volume *32*

Supplemental Information

Small Molecule Enhancers of Endosome-to-Cytosol Import Augment Anti-tumor Immunity

Patrycja Kozik, Marine Gros, Daniel N. Itzhak, Leonel Joannas, Sandrine Heurtebise-Chrétien, Patrycja A. Krawczyk, Pablo Rodríguez-Silvestre, Andrés Alloatti, Joao Gamelas Magalhaes, Elaine Del Nery, Georg H.H. Borner, and Sebastian Amigorena

Figure S1

ERAD inhibitors

Importazole

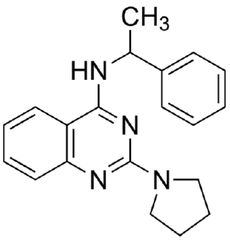

DbeQ

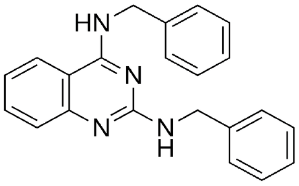

FDA library

Active compounds

Prazosin

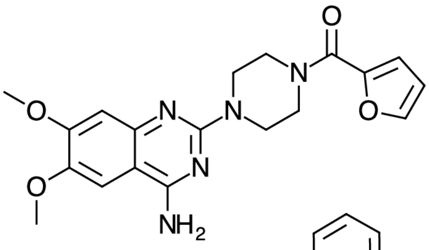

Doxazosin

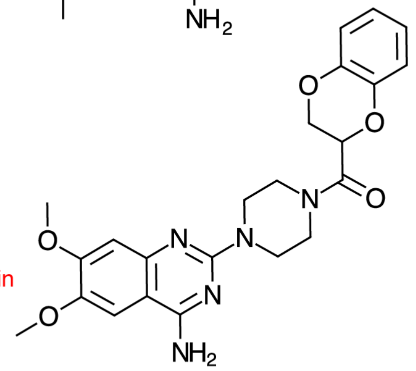

Gefitinib

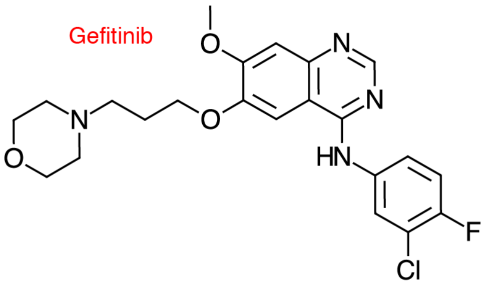

Non-active compounds

Terazosin

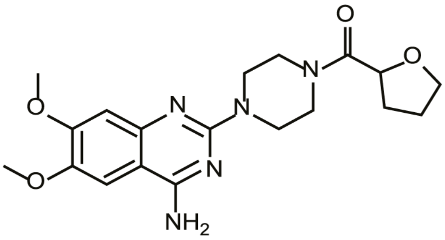

Lapatinib

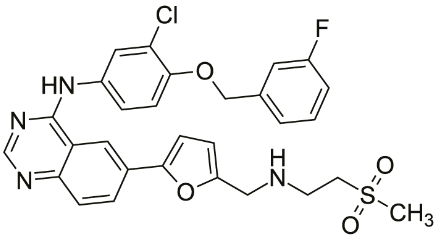

Figure S1. Chemical structures of the ERAD inhibitors and of selected active and non-active quinazolinamine tested in this study.

Related to Figure 1

Figure S2

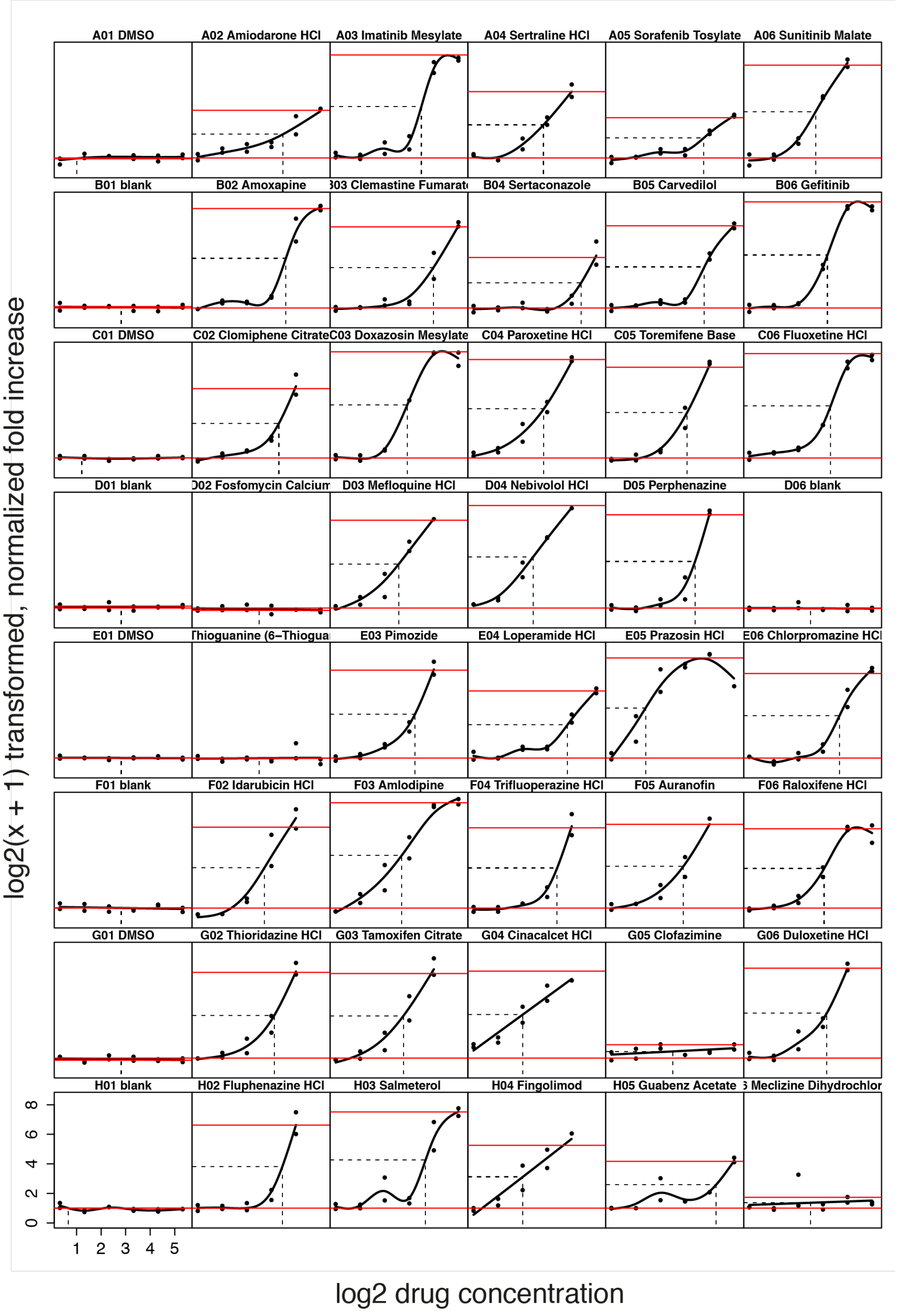

Figure S2. Summary of the EC50 plots from the secondary screen.

Related to Figure 1

39 compounds were analysed using the β-lactamase assay: 37 top ranked compounds from the primary screen and two compounds with no phenotype were included (fosfomycin calcium and thioguanine). No treatment (blank) and vehicle (DMSO) controls were included on each plate. The screen was performed with five doses for each of the drugs (1.25 - 40 μM). Wells with fewer than 500 cells were excluded from the analysis. The proportion of the cells with efficient β-lactamase translocation was determined and these raw phenotype

measurements were normalised by dividing each value by the mean of the "DMSO" control wells from the corresponding plate. drFitSpline function from the grofit R package was used to estimate the yEC50 (50% of the max effect) values. The fold-increase and concentration values were log2(x+1) transformed for spline fitting. The concentration values were log2 transformed. The red lines indicated max and min import efficiency, the horizontal dotted line indicates the yEC50 value and the vertical line the corresponding compound concentration.

Figure S3

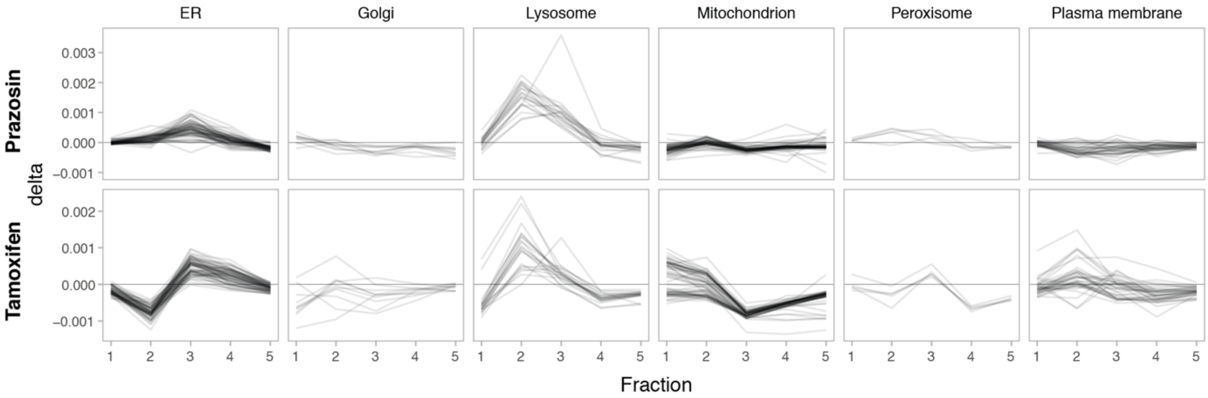

Figure S3. 'Movement' data for proteins from selected organelles used for 'MR' analysis.

Related to Figure 3.

Prazosin- and tamoxifen-induced shifts in protein subcellular localization detected using 'MR' plot analysis. Each line represents the normalised shift for one protein (average from two independent experiments). All proteins assigned to the five organelles represented in the panel are shown.

Figure S4

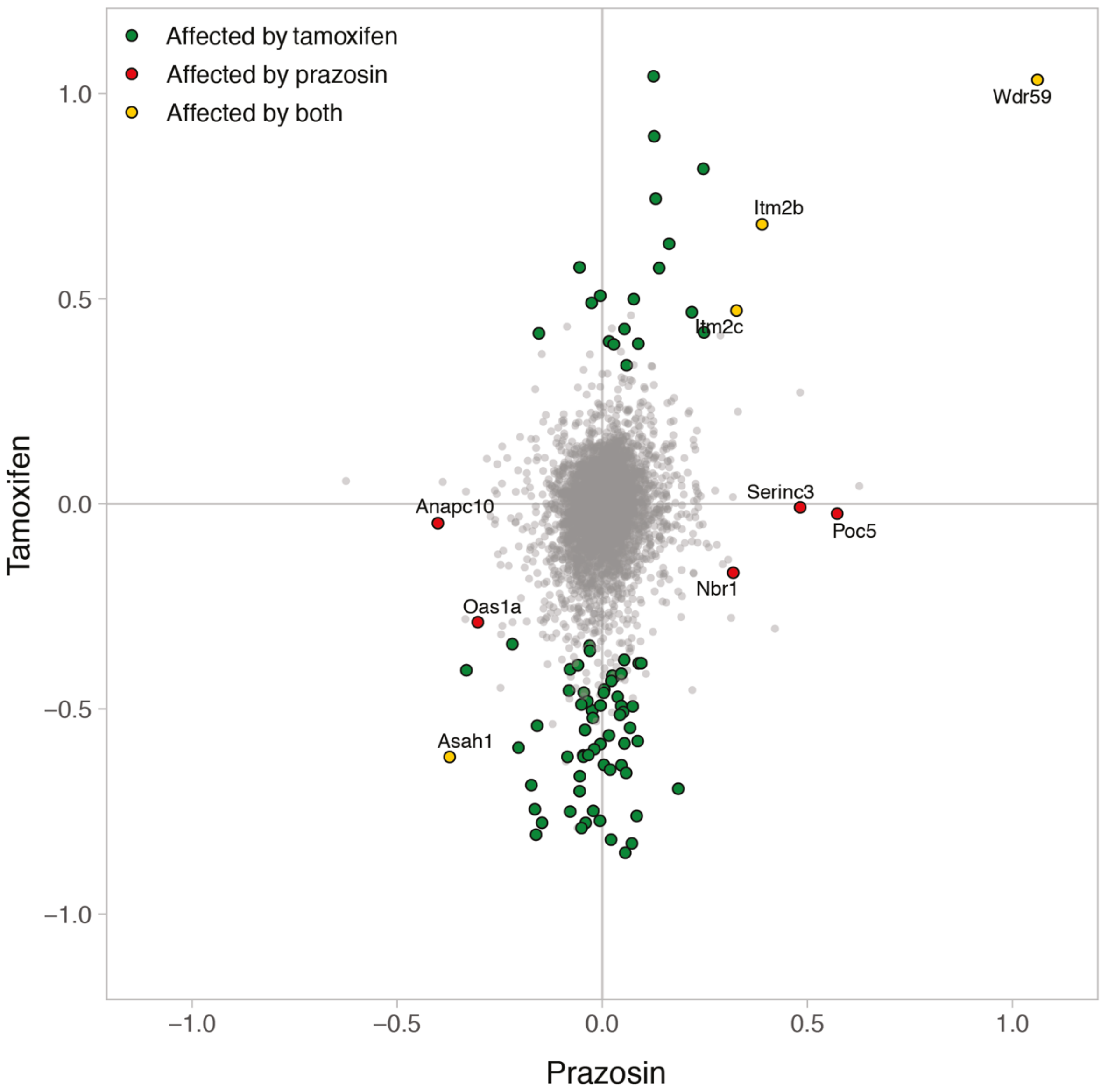

Figure S4. Whole cell protein expression levels in MutuDCs.

Related to Figure 3

Protein expression levels in MutuDCs from prazosin or tamoxifen treated (4 h) cells relative to untreated controls (log 2 scale). Quantification was achieved by metabolic labelling (SILAC; averages of two replicates are shown). 5848 proteins were quantified across all four experiments. Proteins that changed significantly in abundance are highlighted in colour. Tamoxifen treatment induced more changes in proteome composition than prazosin treatment; overall, the changes induced by either treatment were relatively moderate.

Figure S5

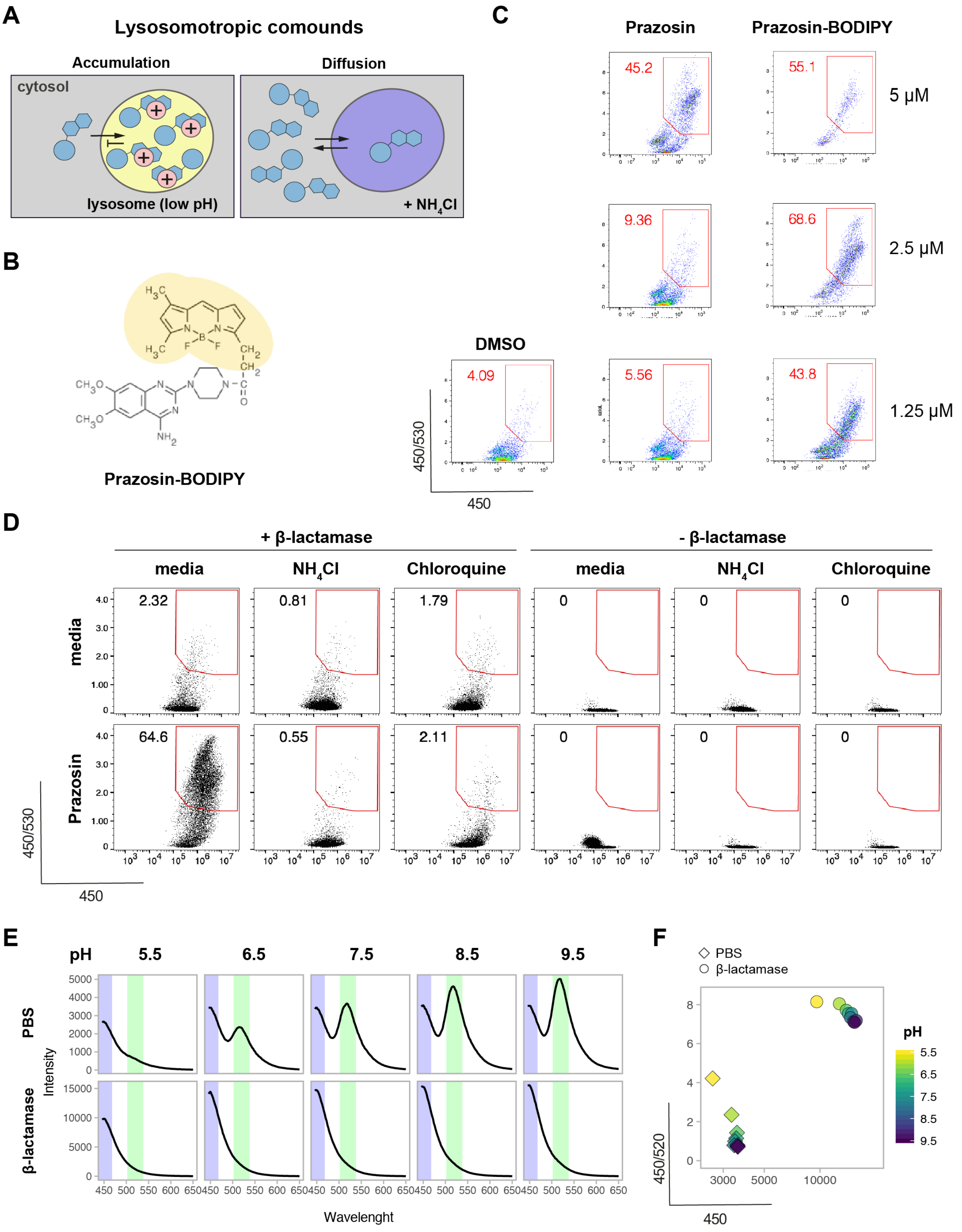

Figure S5. Prazosin activity is linked to accumulation in low pH compartments.
Related to Figure 5

A. Schematic representation of lysosomal trapping. Membrane permeable small molecules diffuse freely across the membrane of acidic compartments. Protonation of weakly basic residues decreases membrane permeability

and leads to accumulation of protonated compounds in the endo-lysosomal lumen. When the pH of intracellular compartments is neutralised, the compounds are no longer protonated and are free to diffuse across the membrane.
B. Comparison of the chemical structures of prazosin and prazosin-BODIPY.
C. Activity of prazosin-BODIPY in the β-lactamase assay. Prazosin modified with BODIPY is active in the antigen import assay.
D. Antigen import assay was performed in the presence of NH_4Cl or chloroquine with or without prazosin. NH_4Cl or chloroquine alone have no effect on antigen import and they both abolish prazosin activity. Cells with the high blue-to-green fluorescence ratio are absent in samples that were not pulsed with β-lactamase.
E and F. To determine whether the β-lactamase assay is sensitive to changes in cytosolic pH, CCF2-FA was incubated in PBS with or without β-lactamase for 90 min (CCF2-FA is a free acid version of the CCF4-AM and it does not require de-esterification by the cytosolic esterases). The fluorescence spectra of cleaved and uncleaved CCF2 were then determined in buffers of different pH using Tecan Spark microplate reader (excitation wavelength: 405 nm). E. Sample spectra for cleaved and uncleaved CCF2. The wavelengths collected during flow cytometry are indicated in blue and green. F. Ratio of blue (emission at 450 nm) to green (emission at 520 nm) fluorescence plotted against fluorescence at 450 nm (similar to the representations for the flow cytometry data). While acidification results in quenching of both CCF4/2 subunits, fluorescein and coumarin, the cleaved and uncleaved dye remain separated.

Figure S6

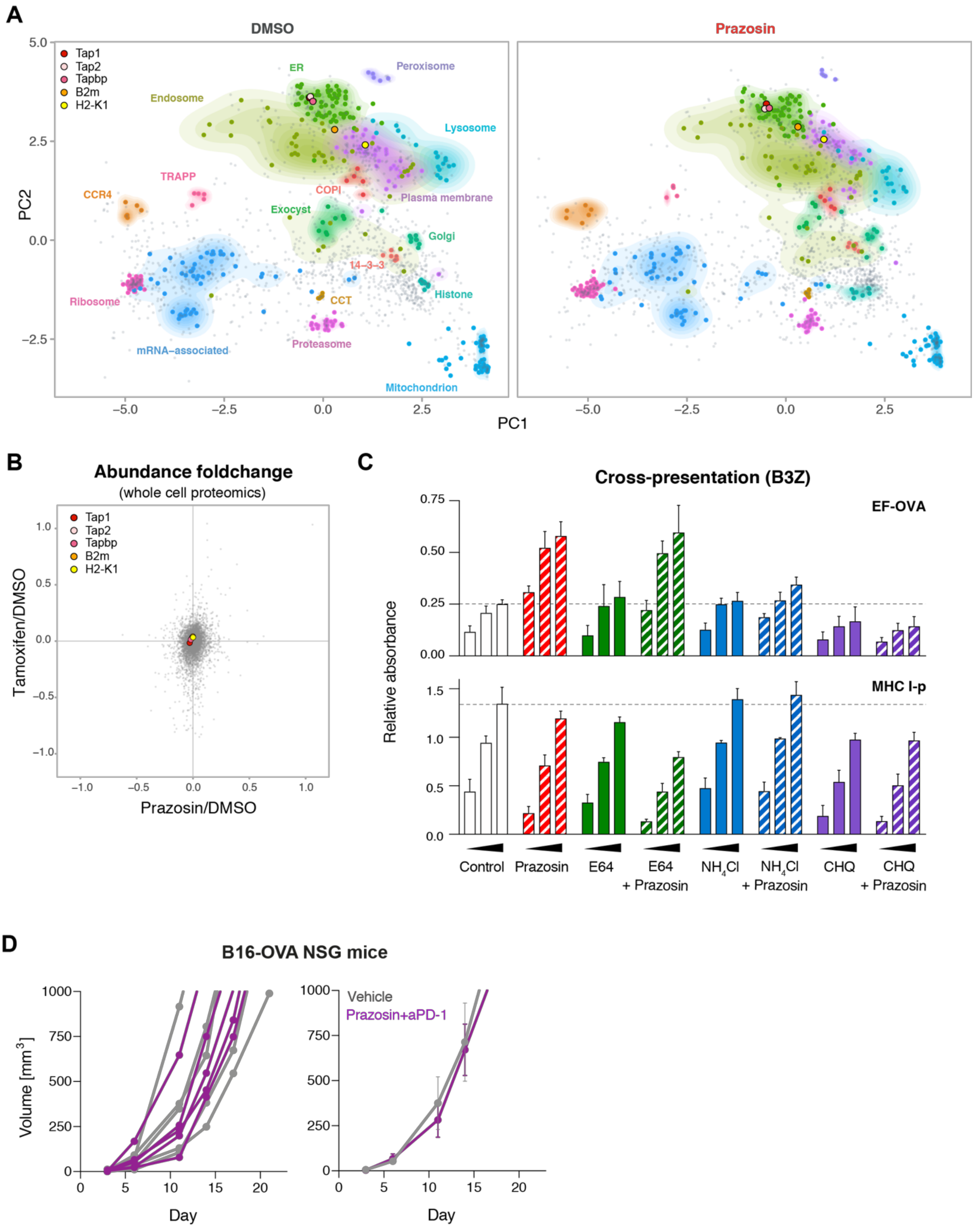

Figure S6. Analysis of the prazosin activity in antigen cross-presentation in vitro and control of tumour growth in vivo.

Related to Figure 6

A. Organellar maps (see Figure 3B) of prazosin- and DMSO-treated cells. Prazosin does not lead to re-localisation of the loading complex or MHC I subunits.

B. Fold increase (log2 scale) of the loading complex components and MHC class I subunits in drug-treated samples (relative to DMSO control) (see Figure S4). Neither prazosin nor tamoxifen lead to a change in abundance of the loading complex components or of the MHC I.
C. MutuDCs were incubated with sOVA EF or MHC I peptide for 5 h, in the presence of indicated compounds. Cross-presentation efficiency was monitored using the B3Z assay. Neutralisation of endolysosomal compartments or inhibition of cathepsins are not sufficient for cross-presentation enhancement in MutuDCs. NH_4Cl and chloroquine abolish the prazosin effect on cross-presentation, similar to the phenotypes observed in antigen import assays.
D. Tumour growth curves for NSG mice injected s.c. with the B16-OVA tumour cells. From the day when tumours became detectable, mice were injected i.p. three times per week with a combination of 0.5 mg prazosin and 150 μg anti-PD-1.
