# Supplementary material for: Small Molecule Enhancers of Endosome-to-Cytosol Import Augment Anti-tumor Immunity
Source: Cell Rep. 2020 Jul 14;32(2):107905. doi: 10.1016/j.celrep.2020.107905 (PMC7370168; doi:10.1016/j.celrep.2020.107905)
Supplement: Document S2. Article plus Supplemental Information [file mmc6.pdf]

Cell Reports

Resource

Small Molecule Enhancers of Endosome-to-Cytosol Import Augment Anti-tumor Immunity

Graphical Abstract

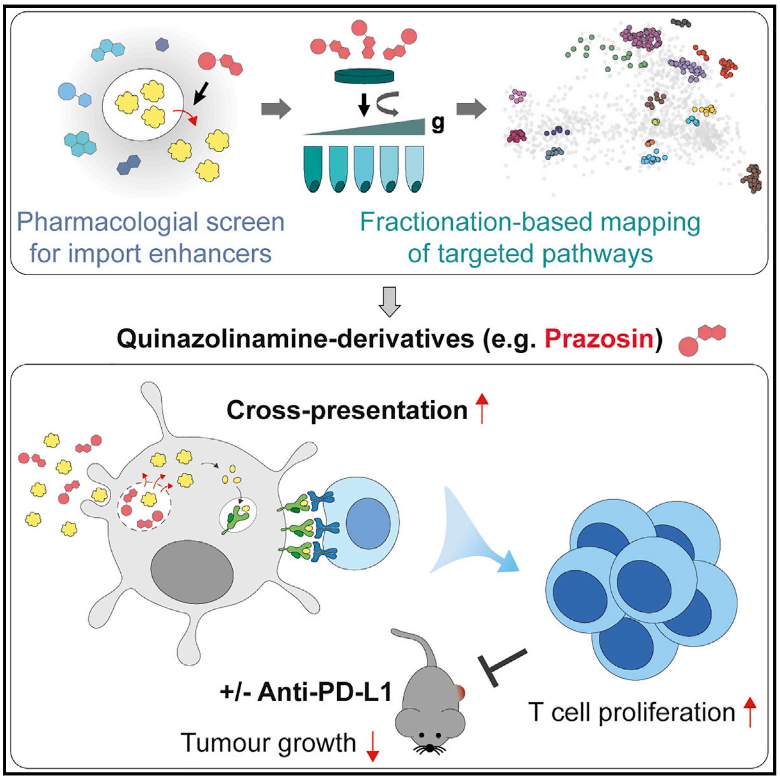

Highlights

- Quinazolinamine derivatives are potent enhancers of antigen import into the cytosol
- Antigen import enhancement occurs as a consequence of lysosomal trapping
- Antigen import enhancers facilitate cross-presentation and synergize with anti-PD-L1
- Proteomic organellar maps can be used as a tool to detect biological effects of drugs

Authors

Patrycja Kozik, Marine Gros, Daniel N. Itzhak, ..., Elaine Del Nery, Georg H.H. Borner, Sebastian Amigorena

Correspondence

pkozik@mrc-lmb.cam.ac.uk

In Brief

Kozik et al. apply a screening strategy to identify drugs that facilitate endosome-to-cytosol import of antigens in dendritic cells during cross-presentation. They characterize selected compounds using proteomic organellar mapping, live-cell imaging, *in vitro* cross-presentation assays, and tumor models and demonstrate that enhancing import facilitates cross-presentation and synergizes with checkpoint-based immunotherapy.

Kozik et al., 2020, Cell Reports *32*, 107905
July 14, 2020 © 2020 The Authors.
https://doi.org/10.1016/j.celrep.2020.107905

CellPress

Cell Reports

CellPress
OPEN ACCESS

Resource

Small Molecule Enhancers of Endosome-to-Cytosol Import Augment Anti-tumor Immunity

Patrycja Kozik,[1,2,5,6,*] Marine Gros,[1] Daniel N. Itzhak,[3] Leonel Joannas,[1] Sandrine Heurtebise-Chrétien,[1] Patrycja A. Krawczyk,[2] Pablo Rodríguez-Silvestre,[2] Andrés Alloatti,[1] Joao Gamelas Magalhaes,[1] Elaine Del Nery,[4] Georg H.H. Borner,[3,5] and Sebastian Amigorena[1,5]
[1]INSERM U932, PSL Research University, Institut Curie, 75005 Paris, France
[2]MRC Laboratory of Molecular Biology, Cambridge CB2 0QH, UK
[3]Max Planck Institute of Biochemistry, 82152 Martinsried, Germany
[4]Institut Curie, PSL Research University, Department of Translational Research-Biophenics High-Content Screening Laboratory, Cell and Tissue Imaging Facility (PICT-IBiSA), 75005 Paris, France
[5]Senior author
[6]Lead Contact
*Correspondence: pkozik@mrc-lmb.cam.ac.uk
https://doi.org/10.1016/j.celrep.2020.107905

SUMMARY

Cross-presentation of antigens by dendritic cells (DCs) is critical for initiation of anti-tumor immune responses. Yet, key steps involved in trafficking of antigens taken up by DCs remain incompletely understood. Here, we screen 700 US Food and Drug Administration (FDA)-approved drugs and identify 37 enhancers of antigen import from endolysosomes into the cytosol. To reveal their mechanism of action, we generate proteomic organellar maps of control and drug-treated DCs (focusing on two compounds, prazosin and tamoxifen). By combining organellar mapping, quantitative proteomics, and microscopy, we conclude that import enhancers undergo lysosomal trapping leading to membrane permeation and antigen release. Enhancing antigen import facilitates cross-presentation of soluble and cell-associated antigens. Systemic administration of prazosin leads to reduced growth of MC38 tumors and to a synergistic effect with checkpoint immunotherapy in a melanoma model. Thus, inefficient antigen import into the cytosol limits antigen cross-presentation, restraining the potency of anti-tumor immune responses and efficacy of checkpoint blockers.

INTRODUCTION

Accumulation of mutations in cancer is a key factor during disease progression, yet, it can also render cancer cells vulnerable to cytotoxic T cells (CTLs). T cell-mediated anti-tumor immune responses are primarily initiated by type 1 conventional dendritic cells (cDC1s) (Böttcher and Reis e Sousa, 2018). Although these immune responses can in principle prevent or restrict tumor growth, they are usually not nearly as potent as responses against pathogens. In recent years, checkpoint inhibitors emerged as a promising tool to enhance anti-tumor immunity and were effective in providing long-lasting remissions. Nevertheless, their efficacy is largely dependent on pre-existing immunity, and the benefits are only seen in a fraction of patients (Crittenden et al., 2018). Therefore, a better understanding of the mechanisms and rate-limiting steps involved in priming of naive tumor-specific T cells will be critical for improving immunotherapeutic strategies.

Efficient priming relies on the delivery of three signals to naive T cells: signal 1, relevant antigen (e.g., a mutated peptide) presented in the context of major histocompatibility complex (MHC) class I; signal 2, co-stimulatory molecules expressed by antigen presenting cells (APCs); and signal 3, cytokines, which ultimately determine whether the response will lead to immunity or tolerance. Many approaches have been explored to deliver appropriate signals 2 and 3, including stimulating DCs maturation with a variety of Toll-like receptor (TLR) agonists (e.g., poly(I:C) or CpG) or growth factors (e.g., FLT3L) (Brunner et al., 2000; Hammerich et al., 2019; Salmon et al., 2016; Sánchez-Paulete et al., 2018). However, increasing the efficiency of presentation of tumor antigens on MHC class I has proven more challenging.

Tumor antigens are presented by APCs via a process termed cross-presentation. Cross-presentation involves endocytic uptake of exogenous proteins followed by generation of short peptides that can be loaded onto MHC class I. Two models have been proposed to describe where peptide generation takes place (reviewed in Gros and Amigorena, 2019). In the vacuolar model, peptides are generated by endolysosomal proteases (primarily cathepsins) and directly loaded onto MHC class I (Shen et al., 2004). In the cytosolic model, antigens are imported into the cytosol, processed by the proteasome, and delivered into the lumen of MHC class I-containing compartments via the TAP transporter (Ackerman et al., 2003; Guermonprez et al., 2003; Kovacsovics-Bankowski and Rock, 1995). Considering differences in cleavage-specificities among the different proteases, the cytosolic model provides an attractive explanation of

© 2020 The Authors.
This is an open access article under the CC BY license (http://creativecommons.org/licenses/by/4.0/).

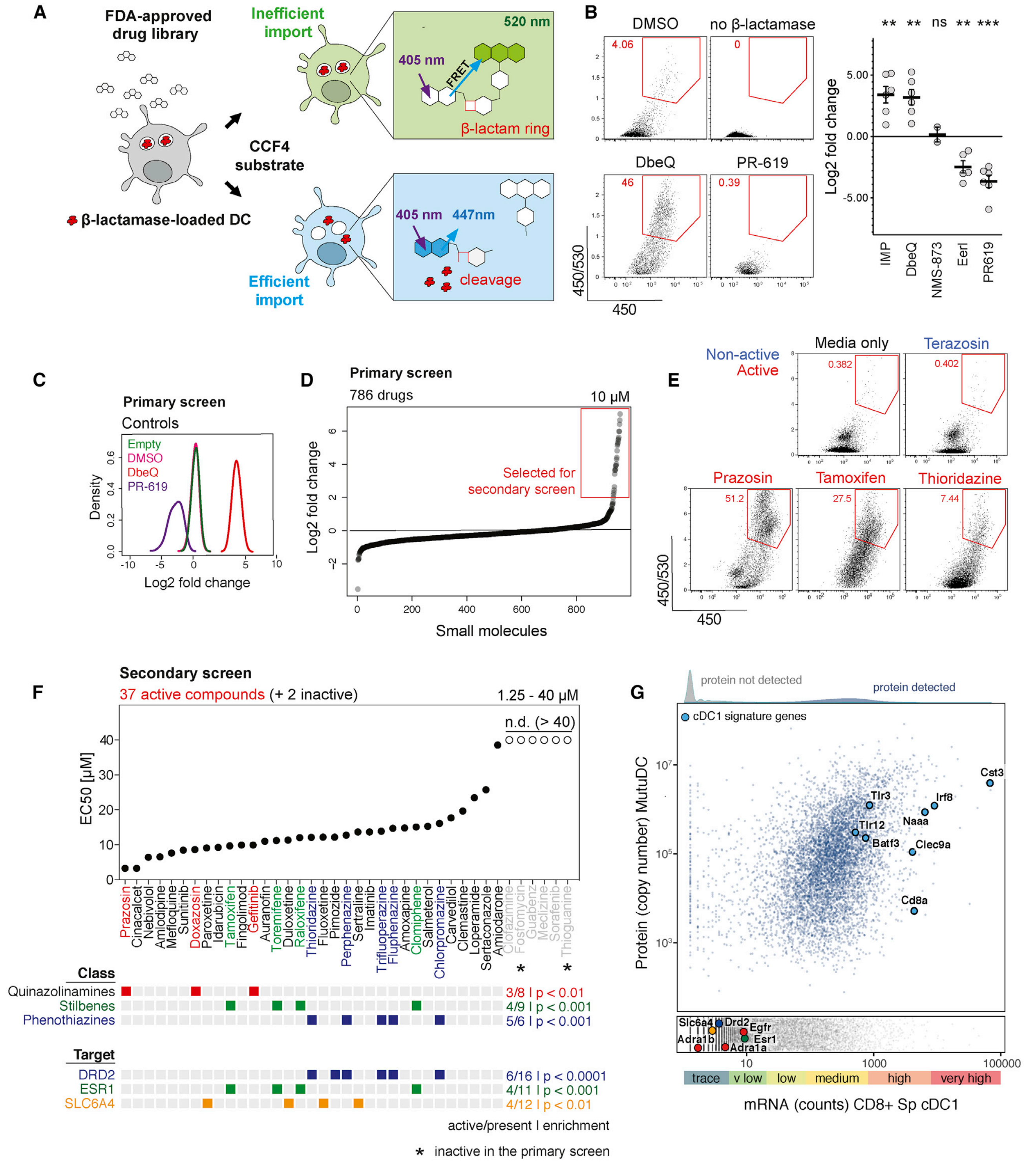

Figure 1. Small Molecule Screen to Identify Enhancers of Antigen Import into the Cytosol

(A) Schematic representation of the β-lactamase assay used to monitor the efficiency of antigen import into the cytosol. MutuDCs were fed with β-lactamase for 3 h followed by 2 h incubation with small molecules (at 37°C). CCF4 loading was performed at room temperature for 1 h, and followed by 16 h incubation at RT to increase the sensitivity of the assay (Zlokarnik et al., 1998). Change in CCF4 fluorescence was monitored by flow cytometry.

(B) Differential effects of ERAD inhibitors on antigen import into the cytosol. Representative flow cytometry data for selected ERAD inhibitors and quantification of the fold change in antigen import relative to DMSO controls. IMP, importazole; EerI, Eeyarestatin I. Means ± SE (dots represents data from independent experiments).

(legend continued on next page)

how APCs would generate peptides similar to those presented by target cells, where the majority of epitopes is also generated by proteasomes. Both TAP- and immunoproteasome-deficient mice are defective in cross-presentation (Palmowski et al., 2006; Rock and Shen, 2005), but whether these effects are indeed due to specific inhibition of cross-presentation, and whether the cytosolic pathway is dominant *in vivo*, still requires verification. Similarly, mechanistic details of endosome-to-cytosol transport have remained elusive.

Irrespective of the precise mechanism, the importance of cross-presentation in initiation of anti-tumor responses has now been demonstrated in a variety of mouse models. cDC1s appear to be most efficient cross-presenters *in vivo* and Batf3$^{-/-}$ mice that lack cDC1s, do not mount efficient T cell responses (Hildner et al., 2008). In mice with a Wdfy4 deletion (Theisen et al., 2018) or a DC-specific knockout of Sec22b (Alloatti et al., 2017), cDC1s are present but deficient in the ability to cross-present. Both models are unable to prime naive T cells against tumor-associated antigens and fail to control tumor growth. Similar to cDC1-deficient mice (Sánchez-Paulete et al., 2016), Sec22b knockouts are also resistant to treatment with checkpoint inhibitors. These data argue for an important role of cross-presentation in anti-tumor immunity. Indeed, delivering tumor antigens to cross-presenting cells (e.g., via antibody-antigen conjugates), has been effective in promoting CTL responses (Bonifaz et al., 2002; Caminschi et al., 2008; Sancho et al., 2008). In the clinic, vaccination with long peptides comprising neoepitopes has also been successfully used to boost generation of tumor-specific T cells (Ott et al., 2017). These approaches of boosting antigen presentation are, however, costly to implement as they require prior identification of cancer neoantigens (e.g., through next generation sequencing of tumor samples).

Here, we present a strategy for enhancing efficiency of T cell priming by facilitating antigen presentation by DCs. Our study was based on the hypothesis that import of internalized antigens into the cytosol might be limiting for the efficiency of cross-presentation. With this in mind, we set up an assay to screen a library of over 700 US Food and Drug Administration (FDA)-approved compounds to identify enhancers of antigen import. We demonstrated that these molecules indeed facilitated cross-presentation of both soluble and cell-associated antigens. To evaluate the biological activity of two import enhancers, prazosin and tamoxifen, we generated comprehensive proteomics-based organellar maps from treated and untreated cells. We established that our most potent compound, prazosin, has a highly specific effect on endolysosomal membrane permeability. This encouraged us to pursue *in vivo* studies, where we demonstrated that systemic administration of prazosin leads to better control of tumor growth and synergizes with checkpoint-based anti-tumor immunotherapy.

RESULTS

Selected Endoplasmic Reticulum-Associated Protein Degradation (ERAD) Inhibitors Enhance Antigen Import

ERAD machinery has been proposed to play a key role in import of antigens from endosomes and phagosomes into the cytosol (Giodini and Cresswell, 2008; Imai et al., 2005; Zehner et al., 2015). Recently, however, we demonstrated that mycolactone, a potent inhibitor of Sec61 (a candidate ERAD translocon), does not inhibit antigen import (Grotzke et al., 2017). Here, we initially employed a pharmacological approach to evaluate the contribution of other ERAD components to antigen import. We selected a range of ERAD inhibitors and tested them using a β-lactamase-based antigen import assay (Figure 1A) (modified from Cebrian et al., 2011). As a model system, we used the cell line MutuDC, which phenotypically corresponds to splenic cDC1s (Fuertes Marraco et al., 2012) (see also Figure 1G). To prevent tested compounds from affecting antigen uptake, we pulsed MutuDCs with β-lactamase for 3 h and subsequently treated them with the different inhibitors for 2 h. To detect β-lactamase translocation into the cytosol, we loaded the cells with a cytosolic β-lactamase substrate, CCF4. When β-lactamase enters the cytosol, it cleaves the β-lactam ring in the CCF4 and disrupts fluorescence resonance energy transfer (FRET) between its two subunits causing a shift in fluorescence from green to blue (Figure 1A). We monitored this change in fluorescence by flow cytometry (Figure 1B). The two compounds that target the ubiquitin pathway, PR-619 and Eeyarestatin I (EerI), inhibited antigen import (Figure 1B, consistent with previous data) (Grotzke et al., 2017; Zehner et al., 2015). Unexpectedly, a p97 inhibitor, DbeQ, and a β-importin inhibitor, importazole, enhanced antigen import

(C) Quality control of the FDA library screen. The histograms show distribution of fold changes in the efficiency of antigen import (relative to DMSO) for each control (all wells across the ten 96-well plates).

(D) Results from the FDA library screen. Fold changes in β-lactamase import for the 786 tested drugs. The screen was performed once, and 37 compounds were selected for the secondary screen (highlighted with the red box).

(E) Examples of the flow cytometry profiles in the antigen import assay for selected active and non-active compounds.

(F) Results from the secondary screen of 37 compounds (and two control compounds, not active in the primary screen). Each drug was tested at five concentrations in two independent experiments. EC_{50} values (concentration required for 50% of maximal activity) values were calculates as described in Figure S2. Information about chemical classes and candidate targets was obtained from the DrugBank database. The classes and targets enriched in the group of active versus non-active compounds are represented with colored squares. The enrichment of targets for hits (compared to the entire library) was calculated using Fisher's exact test.

(G) Analysis of gene and protein expression in CD8$^+$ cDC1s. mRNA expression data (RNA sequencing [RNA-seq]) for CD8$^+$ splenic DCs was downloaded from the www.Immgen.org database (GEO: GSE109125), and whole cell proteomic abundance data were generated by mass spectrometry from MutuDCs. 7427 proteins were detected by proteomics (blue dots) and selected markers highly expressed in cDC1s are highlighted with large blue circles. Absolute copy numbers for all proteins detected in whole cell MutuDC proteome are available via the web resource (http://dc-biology.mrc-lmb.cam.ac.uk). The lower panel (gray dots) includes proteins not detected by proteomics. Targets enriched in the group of active versus non-active compounds are highlighted (Esr1, Drd2, and Slc6a4), as well as targets of the three active quinazolinamines (Adra1b for prazosin, Adra1a for doxazosin, and Egfr for gefitinib).
See also Figures S1 and S2 and Data S1 and S2.

(Figures 1B and S1). This effect was not recapitulated with a more potent p97 inhibitor, NMS-873, suggesting it might be due to off-target activity. Hence, although these data highlight the role of the ubiquitin system in antigen import, they did not provide evidence supporting the role of other ERAD components. The dramatic enhancement of antigen import observed with two of the compounds suggests that antigen import is relatively inefficient, and that it may be rate-limiting for cross-presentation.

β-Lactamase-Based Screen for Enhancers of Antigen Import

Enhancement of antigen import by DbeQ and importazole established a proof of concept that this process can be pharmacologically manipulated and prompted us to develop a screen for small molecule import enhancers. We performed the screen in MutuDCs using the β-lactamase-based antigen import assay and a library of 786 FDA-approved drugs (Data S1). DbeQ and PR-619 were used as controls on each plate to track data quality (Figure 1C). We selected 37 drugs that increased antigen import at least 2-fold in the primary screen for follow up (Figures 1D and 1E). Two non-active compounds were also included as additional negative controls (fosfomycin and thioguanine). 32 out of the 37 compounds exhibited a dose-dependent effect in the secondary screen (86% validation rate, 4% hit rate) (Figures 1F and S2). They included three classes of chemically related compounds: quinazolinamines (prazosin, doxazosin, and gefitinib), stilbenes (clomiphene, raloxifene, tamoxifen, and toremifene), and phenothiazines (chlorpromazine, fluphenazine, perphenazine, thioridazine, and trifluoperazine) (Figures 1F and S1).

To understand the mechanism of antigen import enhancement, we first searched for common targets among the active compounds. Using the DrugBank database (Law et al., 2014), we identified previously described targets for 714 of the compounds present in the FDA library. Three of these targets were significantly enriched among active versus non-active compounds: estrogen receptor (Esr1), dopaminergic receptor (Drd2), and serotonin transporter (Slc6a4) (Figure 1F, lower panel). However, none of these three proteins is actually expressed in $CD8^+$ cDC1s according to Immunological Genome Project expression data (Yoshida et al., 2019); they are also not present among the 7,427 proteins we detected in MutuDC by proteomics (Figure 1G; Data S2). Considering that out of 11 estrogen receptor modulators present in the library, antigen enhancement was only observed for inhibitors from the stilbene family, the enrichment appeared to be linked to the structure of these compounds, rather than to the inhibition of known targets. Similarly, no protein and only trace mRNA were detected for targets of the most potent class of enhancers identified, quinazolinamines (Adra1, Adra2, and Egfr). Interestingly, DbeQ and importazole also belong to the quinazolinamine family; hence, half of the ten quinazolinamine derivatives tested in this study facilitated import of internalized antigens, despite being marketed as inhibitors of different targets.

Organellar Maps to Determine Biological Activity of Small Molecules in DCs

A variety of "hidden phenotypes" and promiscuous effects have been observed for numerous clinically approved drugs (MacDonald et al., 2006). These additional phenotypes can often be beneficial for novel therapeutic indications, yet there are few approaches to detect the cellular effects of a compound in an unbiased manner. To characterize the mechanism of antigen import enhancement, we developed a generic strategy to evaluate the biological activity of pharmacological compounds through comparative spatial proteomics (Figure 2A). Many, if not most, cell biological processes are accompanied by protein subcellular localization changes (Lundberg and Borner, 2019; Borner, 2020). Hence, we adapted our previously developed method for generating organellar maps to pinpoint the subcellular localizations of thousands of proteins in a single experiment (Itzhak et al., 2016, 2017, 2019). The comparison of organellar maps made under different physiological conditions allows the capture of drug induced protein translocations (Itzhak et al., 2016, 2017) and thus provides a universal and scalable tool for inferences about cellular responses and drug targets.

To generate organellar maps, we separated post-nuclear supernatants from MutuDCs into five pellets obtained by differential centrifugation (Figure 2A). Each pellet was mixed 1:1 with a SILAC heavy "reference" membrane fraction, and the samples were analyzed by mass spectrometry (MS). To generate abundance profiles, we calculated the heavy-to-light ratio for each protein in each fraction. Using organellar markers we previously established for HeLa cells (Itzhak et al., 2016), we confirmed clustering of proteins from different organelles (e.g., lysosome, peroxisome, and mitochondria) and protein complexes (e.g., proteasome and CCR4-NOT complex) (Figures 2B and 2C). These maps cover over 2,000 proteins expressed in DCs and can be mined for protein subcellular localization, absolute abundance (copy numbers and cellular concentrations), as well as nearest neighbors (i.e., potential interaction partners) via a web resource (http://dc-biology.mrc-lmb.cam.ac.uk; Data S2).

We focused on two import enhancers from different chemical classes, prazosin (quinazolinamine) and tamoxifen (stilbene). To investigate their effects on organellar dynamics, we prepared maps from drug or vehicle-treated MutuDCs in biological duplicates (six maps in total; Data S3). To detect significant protein translocations, maps of control and drug-treated cells were compared using MR (movement and reproducibility) plot analysis (Figures 3A and S3). Tamoxifen treatment led to spatial rearrangements of 56 proteins in MutuDCs, whereas prazosin affected only 33 proteins. The majority of prazosin hits (27/33) mapped to the lysosomal compartment (Figure 3B). These hits comprised 23 out of 24 detected soluble lysosomal enzymes (e.g., cathepsins) as well as three transmembrane proteins. Out of 13 proteins shifting with both drug treatments, 12 also mapped to the lysosome (for other lysosomal proteins, the movement [M] scores in the tamoxifen sample were just below the threshold). Other proteins that shifted upon tamoxifen treatment included components of COPI vesicles, stress granules (e.g., Caprin1 and G3bp1), or CCR4-NOT complex (Figure 3B; Data S3). Thus, in dendritic cells, tamoxifen has pleiotropic effects and prazosin is highly specific, but there is a common effect of both compounds on lysosomal proteins.

We went on to analyze the overall behavior of lysosomal proteins detected in MutuDCs in more detail. While the majority of soluble lysosomal proteins had high M scores (shift to the right

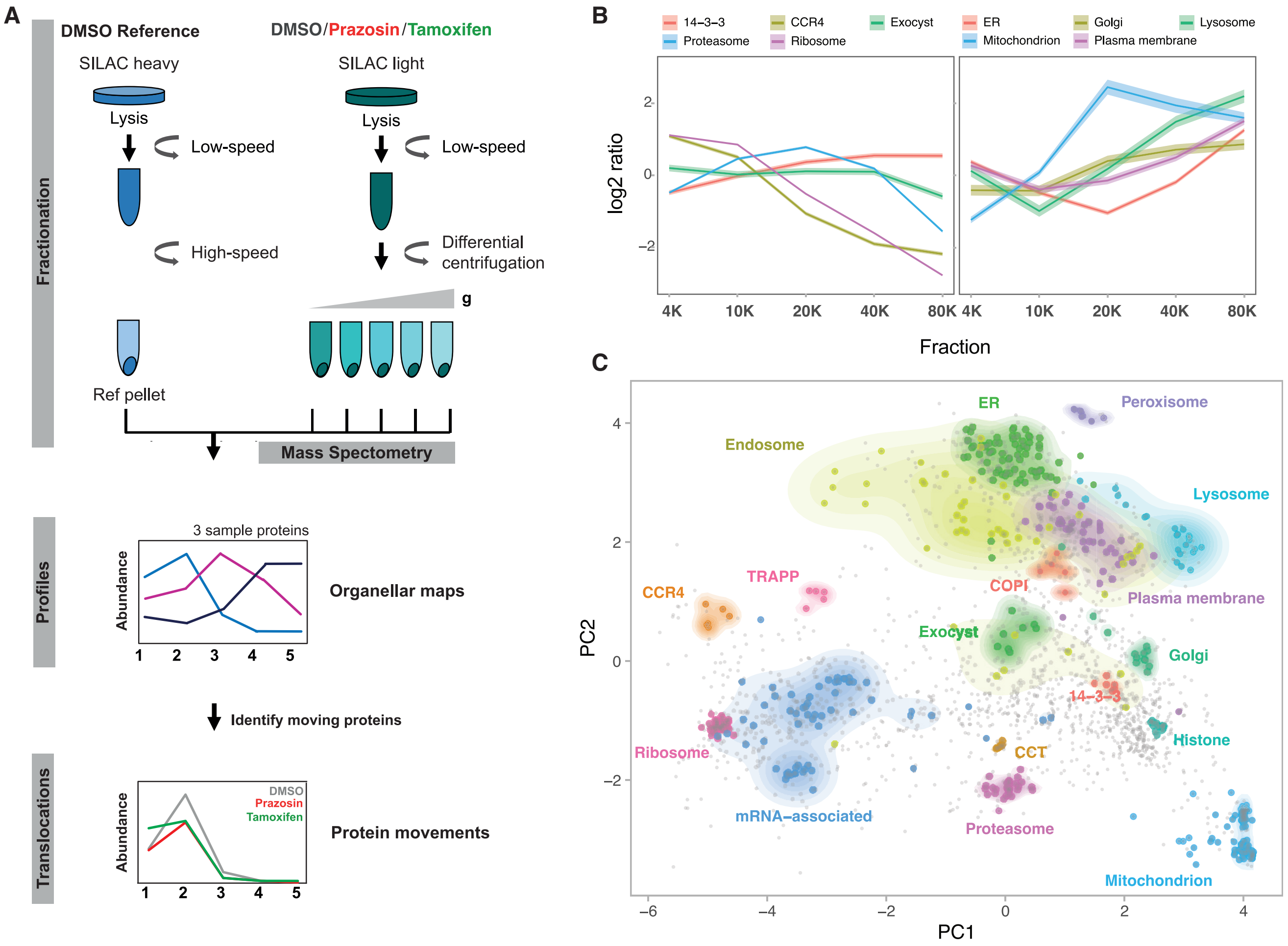

Figure 2. Organellar Mapping in Dendritic Cells

(A) Schematic representation of the fractionation profiling approach for making organellar maps. Metabolically labeled (SILAC heavy—vehicle-treated and light—vehicle- or drug-treated) MutuDCs are lysed mechanically. Post-nuclear supernatant from light labeled cells is subjected to a series of differential centrifugation steps to separate organelles partially. In parallel, post-nuclear supernatant from heavy labeled cells is pelleted at high speed to obtain a reference fraction, which is spiked into each of the light fractions. Quantitative mass spectrometry allows the accurate determination of abundance distribution profiles across the light subfractions for individual proteins. Proteins associated with the same organelle have similar profiles, and different organelles have distinct profiles. Principal component analysis is used to visualize organellar clusters.

(B) Examples of the log2 heavy/light ratios for proteins in selected organelles and protein complexes from vehicle treated MutuDCs (mean ± 95% confidence interval [CI]).

(C) Organellar maps of MutuDCs visualized by principal component analysis. The first two principal components account for >90% of the variability in the data. Marker proteins of various organelles and known protein complexes are shown with colored circles; density gradients for proteins in each cluster are also highlighted.

See also Data S2.

of the MR plot), lysosomal transmembrane proteins show little or no translocation (Figure 3C). This difference in behavior of soluble and transmembrane proteins suggests that lysosomal contents are either secreted into the extracellular space or leaked into the cytosol.

Prazosin and Tamoxifen Induce Lysosome Permeability

To determine whether lysosomal contents in prazosin- and tamoxifen-treated cells are secreted or leaked, we performed quantitative proteomic analyses of whole cell extracts and cytosolic fractions (Data S3). We observed significantly elevated levels of lysosomal enzymes in the cytosol of prazosin and tamoxifen-treated MutuDCs relative to control cells (Figure 4A). Because the levels of these proteins were not changed in whole cell proteome (Figures 4A and S4), we concluded that both prazosin and tamoxifen facilitate lysosomal leakage. Similar to what we observed using organellar maps, the prazosin effect is mostly restricted to lysosomal proteins, whereas tamoxifen affects a larger and more diverse set of proteins.

To rule out that the observed lysosomal leakage was caused by increased compartment fragility and enhanced rupture during cell fractionation, we tested permeability of endolysosomal

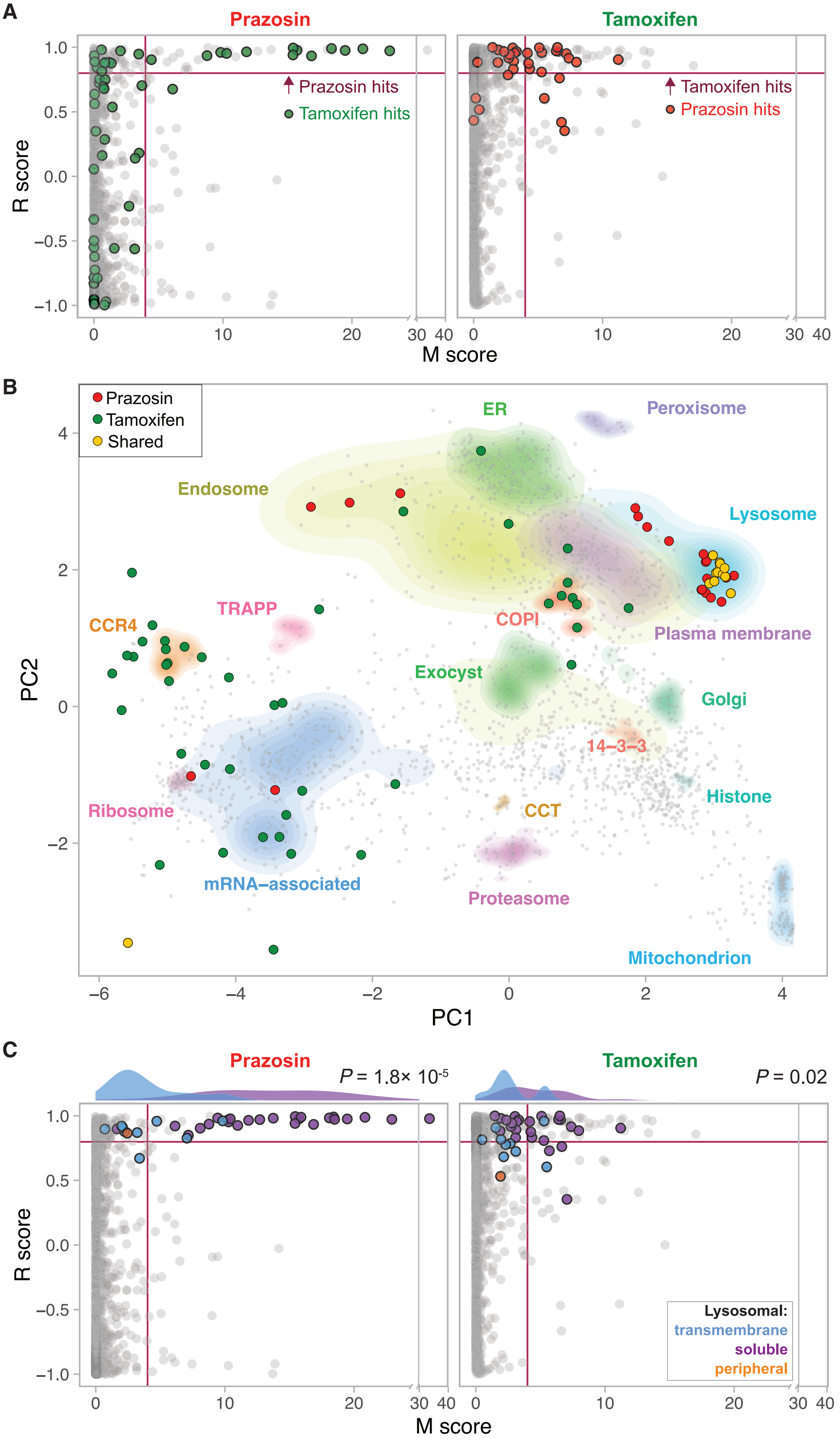

(legend on next page)

compartments in live cells. To this end, we used galectin-3-YFP probe and video microscopy. Galectin-3 is a cytosolic protein that associates with the carbohydrates on the luminal side of the endolysosomal compartments when membranes are damaged (Thurston et al., 2012). In control cells, galectin-3 signal is diffuse, and galectin-3-positive structures are rarely observed (Figure 4B; Video S1). Following addition of prazosin, however, there are frequent bursts of galectin-3-YFP recruitment to vesicular and tubular compartments in MutuDCs (Figures 4B and 4C; Video S1). We also pulsed MutuDC with fluorescent dextran and subsequently treated them with prazosin or tamoxifen. In control cells, dextran remained contained within endolysosomal compartments; in prazosin- and tamoxifen-treated cells, we observed dextran leakage into the cytosol (Figures 4D and 4E). The accessibility of the endolysosomal lumen to a cytosolic galectin-3 probe, as well as release of internalized dextran into the cytosol, demonstrates that in prazosin-treated cells endolysosomal membranes become permeable. In summary, we conclude that both prazosin and tamoxifen target endocytic compartments, causing membrane destabilization and leakage of contents, including internalized antigens, into the cytosol.

Lysosomotropic Properties of Quinazolinamines Mediate Import Enhancement

Considering that in dendritic cells, prazosin had a highly specific effect on lysosome permeability, we hypothesized that the enhancement of antigen import might be mediated through lysosomal trapping of the drugs. Lysosomal trapping occurs when a compound readily crosses membranes at neutral pH, but becomes protonated and membrane impermeable at acidic pH (Figure S5A). This phenomenon has been observed for several classes of amine group-containing, amphiphilic compounds (Nadanaciva et al., 2011). Interestingly, the majority of the hits have physicochemical properties of lysosomotropic compounds, i.e., a pKa between 6.5 and 11 and a logP value >2 (Figure 5A). We used BODIPY-conjugated prazosin to determine whether prazosin undergoes lysosomal trapping (Figures S5B and S5C). Indeed, within seconds following addition, prazosin-BODIPY rapidly accumulated in vacuolar compartments in MutuDCs, positive for fluorescently labeled wheat germ agglutinin (WGA) (Figure 5B). This localization of the dye was dependent on the prazosin moiety, as BODIPY alone (that stains lipid droplets) did not colocalize with WGA (Figure 5B). As predicted, accumulation of prazosin-BODIPY was dependent on the low pH of the endolysosomal compartments and was greatly diminished in cells pretreated with NH_4Cl (Figures 5C and 5D).

To test whether lysosomal trapping of the compounds is required for antigen import enhancement, we performed the β-lactamase import assay in the presence and absence of NH_4Cl to neutralize the lysosomal pH. For all four compounds tested (prazosin, tamoxifen, DbeQ, and importazole), the enhancement of β-lactamase import was completely abolished in the presence of NH_4Cl (Figures 5E and S5D). Intriguingly, neither NH_4Cl nor chloroquine alone enhanced antigen import in MutuDCs, suggesting that dissipation of endolysosomal pH is not sufficient for import enhancement (Figures S5D–S5F). NH_4Cl also abolished prazosin-mediated release of dextran into the cytosol (Figures 5F and 5G). Together, these data support the model where endolysosomal accumulation of the import enhancers leads to destabilization of the antigen-containing compartments.

Antigen Import Enhancers Augment Cross-Presentation and Cross-Priming

To determine if enhanced antigen import results in increased antigen cross-presentation to $CD8^+$ T cells, we fed DCs with soluble ovalbumin (sOVA) prior to treatment with the drugs and incubation with $K^b/OVA_{257–264}$-specific B3Z T cell hybridoma. Both prazosin and tamoxifen treatment led to a dramatic, concentration-dependent enhancement of B3Z activation (Figure 6A). We observed a similar enhancement of cross-presentation of cell associated antigens (Figure 6B); in these experiments we co-cultured 3T3s (H2-K^d) expressing cytosolic OVA with MutuDCs for 5 h in the presence or absence of prazosin, and added the B3Z hybridomas to fixed co-cultures. As demonstrated using a membrane labeling dye (PKH-26^+), prazosin did not increase uptake of cell-associated material (Figure 6C). Importantly, we were also able to enhance cross-presentation of endotoxin-free sOVA (Figure 6D), which is normally not cross-presented efficiently due to the absence of TLR agonists (Alloatti et al., 2016; Burgdorf et al., 2008). This enhancement was not due to prazosin-mediated DC activation, because we did not observe upregulation of activation markers in prazosin-treated DCs (Figure 6E). Prazosin also did not lead to changes in localization or abundance of MHC class I or of the components of the loading complex (Figures S6A and S6B). Importantly, prazosin did not enhance T cell activation when DCs were pulsed with the short $OVA_{257–264}$ peptide (that binds to MHC I without the need for intracellular processing), indicating that it does not affect the general ability of DCs to activate T cells (Figures 6F and S6C). Finally, in accordance with the proposed mechanism of prazosin action, we did not observe an increase in cross-presentation enhancement when prazosin was added in the presence of

Figure 3. Dynamic Organellar Mapping to Identify the Subcellular Changes in Protein Distribution on Drug Treatment

MutuDCs were treated with either prazosin, tamoxifen, or DMSO (control) for 4 h in biological duplicate, and samples were processed as described in Figure 2A. Statistical comparison of organellar maps made with different treatments was performed to identify proteins with profile shifts/altered subcellular localization.

(A) Drug-induced shifts in protein subcellular localization detected using a "MR" plot analysis. For each protein, the movement (M score) and the reproducibility of the movement (R score) between maps was determined. Purple lines indicate cut-offs for significance. In the prazosin plot the hits from tamoxifen treatment are shown for comparison (and vice-versa). Most prazosin hits are also tamoxifen hits or near-hits.

(B) Shifting proteins from tamoxifen and prazosin-treated samples represented on the organellar map of MutuDCs. Most shared hits are lysosomal proteins.

(C) MR plot highlighting all detected lysosomal proteins (soluble, transmembrane, and peripheral [located on the cytosolic side of the membrane]). The histograms show distribution of the M scores for transmembrane and soluble lysosomal proteins.

p values were calculated using the Mann-Whitney U test. See also Figure S3 and Data S2.

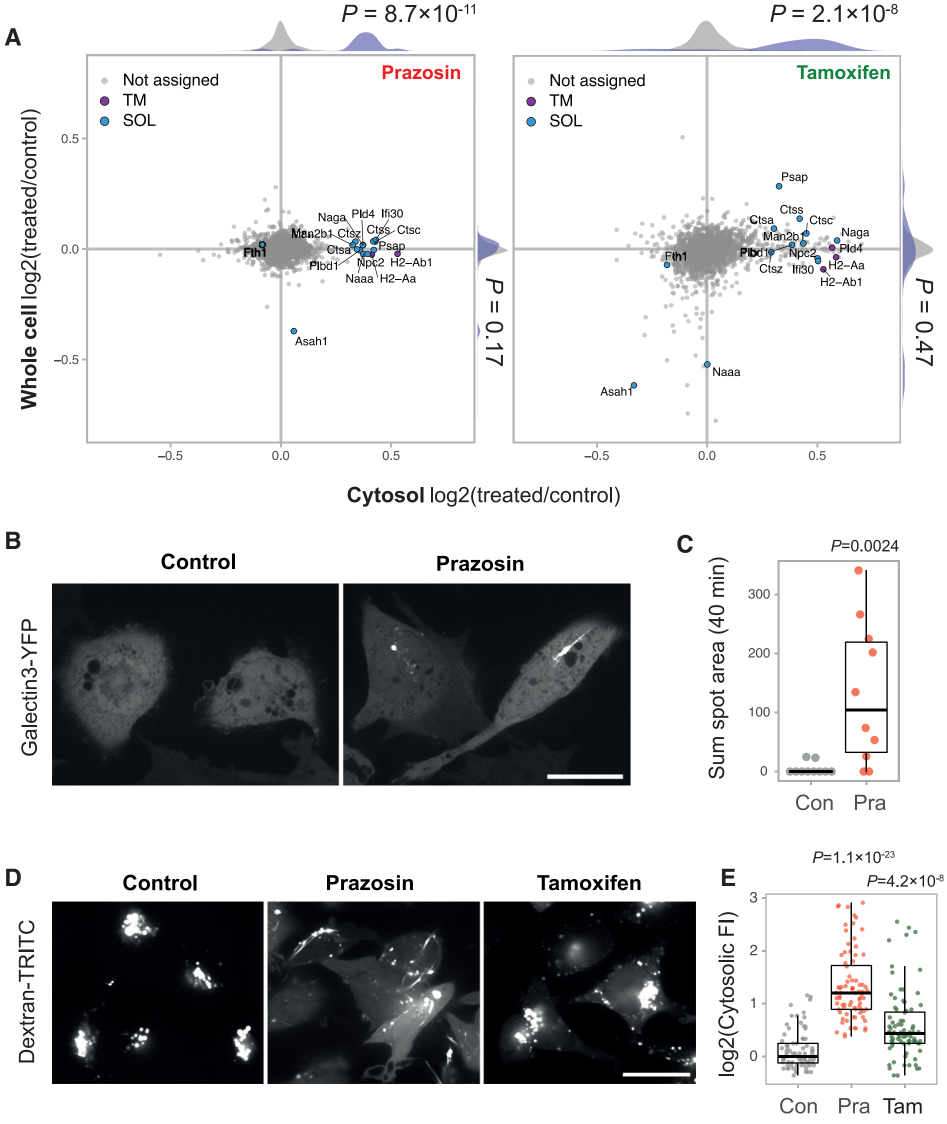

Figure 4. Prazosin and Tamoxifen Lead to Lysosomal Leakage

(A) Analysis of whole cell proteome and cytosol fractions from MutuDCs treated with prazosin, tamoxifen, or DMSO (control) for 4 h. The relative abundance of proteins from prazosin or tamoxifen versus vehicle-treated cells in whole cell versus cytosol proteomes. The histograms show distributions of all (gray) versus lysosomal (purple) proteins. TM, transmembrane; SOL, soluble. p values were calculated using the Kolmogorov-Smirnov test. n = 2 for whole cell lysates (SILAC quantification), n = 4 for cytosol fractions (label-free quantification).

(B and C) MutuDCs stably expressing galectin-3-YFP were treated with 20 μM prazosin and imaged continuously for 40 min.

(B) Examples of control and prazosin-treated galectin-3-YFP-positive cells. Scale bar, 20 μm.

(C) Quantification of galectin-3-YFP recruitment in control and prazosin-treated cells from a representative movie. Each dot represents a sum of galectin-3-YFP spot areas per cell over the duration of the movie (40 min). Box and whiskers plot visualizes median, first, and third quartiles (hinges) and the smallest/largest observation no further than 1.5 * IQR (interquartile range) from the respective hinge (whiskers). p values were calculated using Mann-Whitney U test. Data representative of two experiments.

(D and E) MutuDCs were pulsed with 1 mg/mL 3K dextran-TRITC for 45 min and treated with 20 μM prazosin or tamoxifen for 1 h.

(D) Representative images. Scale bar, 20 μm.

(E) Quantification of the cytosolic fluorescence. Each dot represents a cell (three samples per condition). p values were calculated using Mann-Whitney U test with Bonferroni correction (comparing with the control group). Box and whiskers plot visualizes median, first, and third quartiles (hinges) and the smallest/largest observation no further than 1.5 * IQR from the respoective hinge (whiskers).

See also Figure S4 and Video S1.

CellPress
OPEN ACCESS

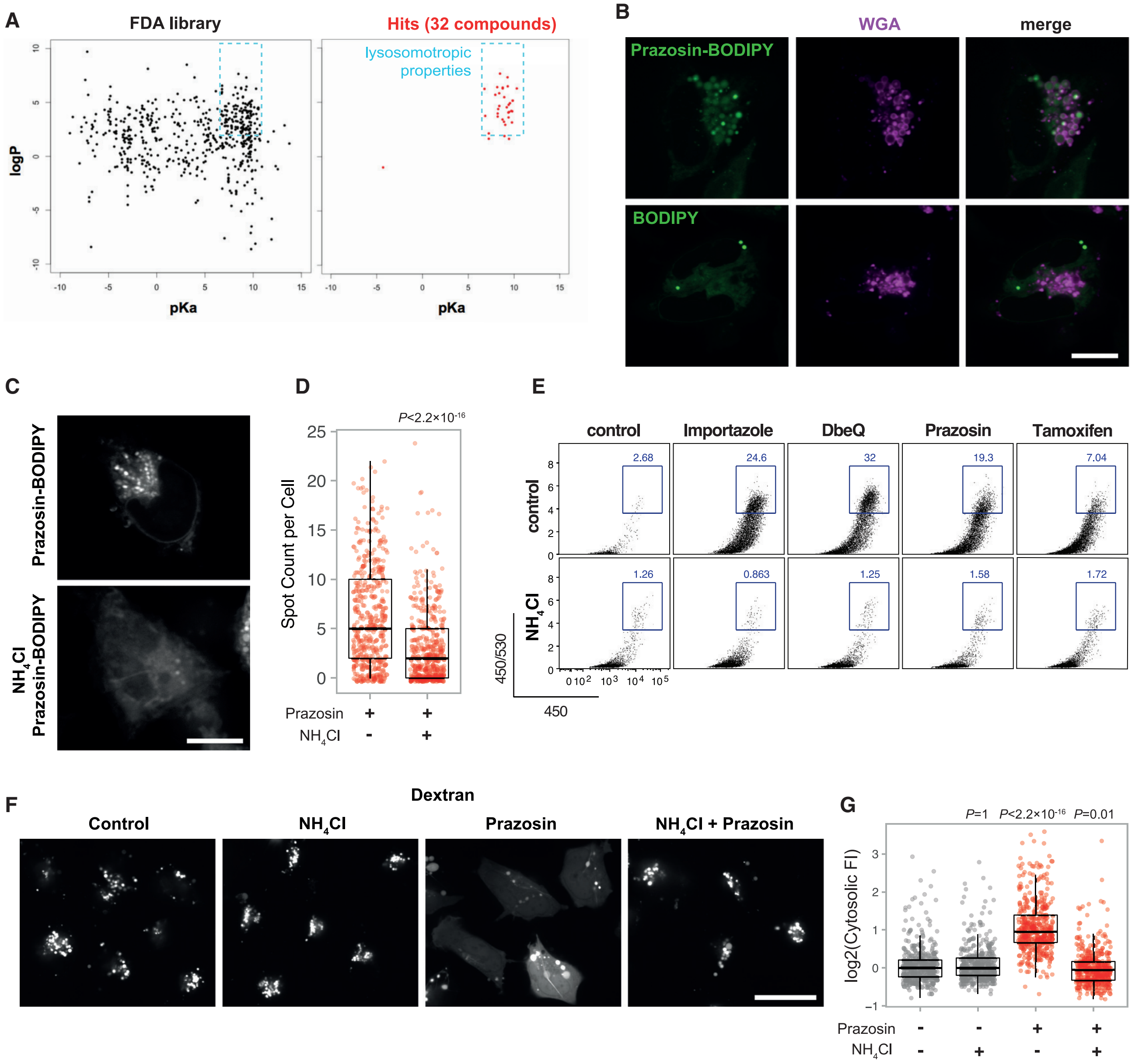

Figure 5. Antigen Import Enhancement Is a Consequence of Lysosomal Trapping

(A) Physicochemical properties (according to DrugBank data) of all the compounds present in the FDA library and of those active in the antigen import assay (yEC$_{50}$ <40 μM, see Figure 1F). All but one of the hits has physicochemical properties similar to those of lysosomotropic compounds (see also Figure S5A).

(B) MutuDCs cells were pulsed with 10 μg/mL WGA-Alexa Fluor 633 for 45 min and imaged immediately after addition of 5 μM prazosin-BODIPY or 10 μg/mL BODIPY. Representative data from one of two independent experiments. Scale bar, 10 μM.

(C and D) MutuDCs were pre-treated with 10 mM NH_4Cl and imaged immediately following addition of prazosin-BODIPY.

(C) Representative images, scale bar 10 μM.

(D) Number of prazosin-BODIPY spots per cell (80 cells per condition per experiment, three independent experiments). p value was calculated using Mann-Whitney U test. Box and whiskers plot visualizes median, first and third quartiles (hinges) and the smallest/largest observation no further than 1.5 * IQR from the respective hinge (whiskers).

(E) Antigen import assay (Figure 1A) was performed in the presence of prazosin, importazole, tamoxifen, and DbeQ with or without NH_4Cl. Representative plots are shown (prazosin, n = 3; tamoxifen, importazole, and DbeQ, n = 2).

(F and G) MutuDCs were pulsed with 3K dextran-TRITC for 45 min and incubated with 20 μM prazosin, 10 mM NH_4Cl, or both for 45 min.

(F) Representative images; scale bar, 20 μM.

(G) Quantification of cytosolic fluorescence. Each dot represents one cell; fluorescence was quantified for 100 cells per condition per experiment with four independent experiments (including data in Figure 4E). p values were calculated using Mann-Whitney U test with Bonferroni correction (comparing with the control group). Box and whiskers plot visualizes median, first, and third quartiles (hinges) and smallest/largest observation no further than 1.5 * IQR from the respective hinge (whiskers). See also Figure S5.

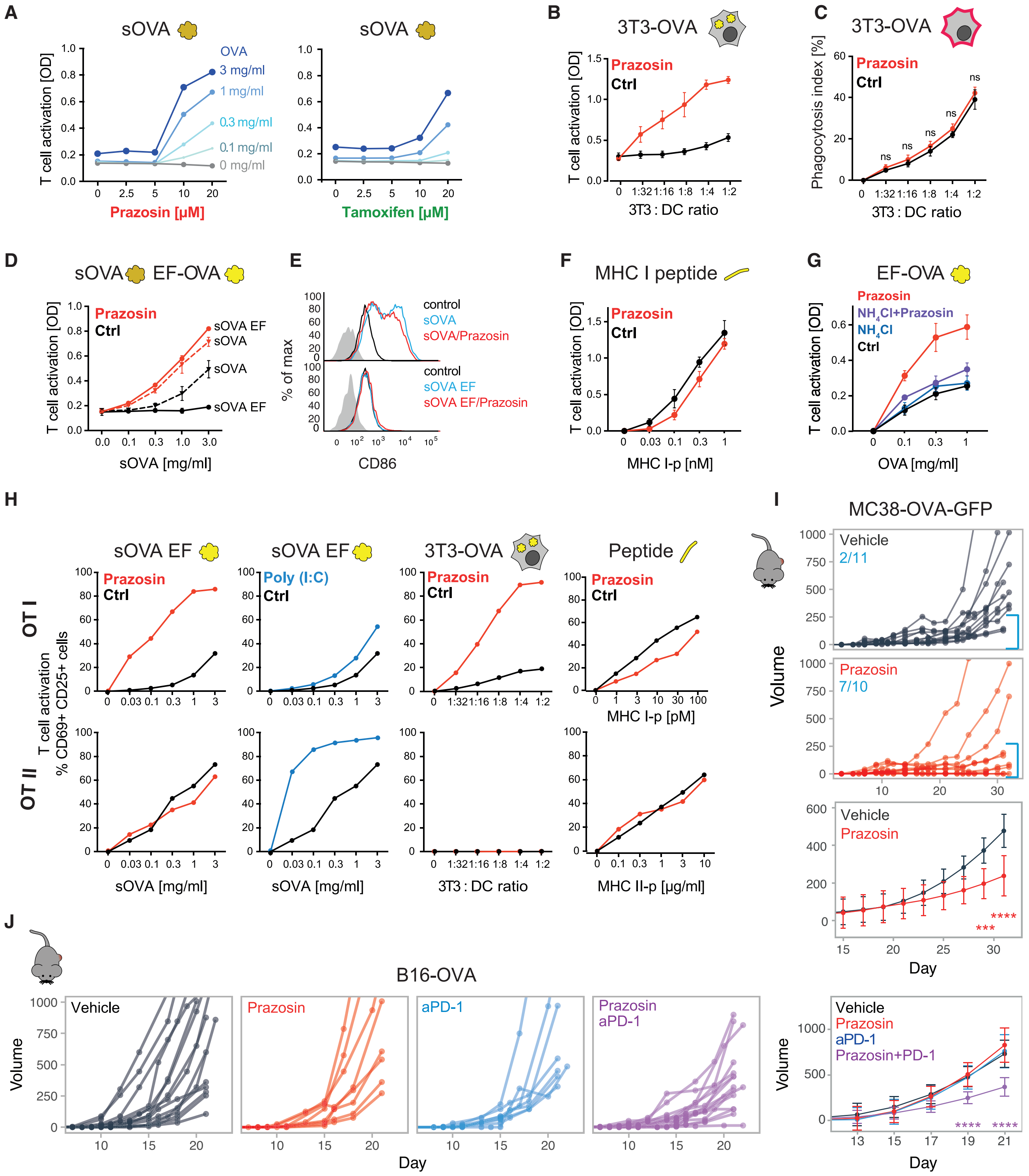

Figure 6. Prazosin Enhances Cross-Presentation and Cross-Priming

(A) Antigen cross-presentation assay with B3Z hybridoma in the presence of increasing concentrations of prazosin or tamoxifen. The cells were pulsed with sOVA for 45 min, followed by 3.5 h incubation in the presence of the indicated compounds. Representative of three independent experiments.

(B) The effect of prazosin on cross-presentation of cell-derived antigens. 3T3 cells expressing cytosolic OVA were used as antigen source and co-cultured with MutuDCs in for 5 h in the presence or absence of prazosin. Mean from three independent experiments ±SE.

(legend continued on next page)

NH_4Cl or chloroquine suggesting endolysosomal accumulation of prazosin is required for the observed phenotype (Figures 6G and S6C). Considering that inhibiting lysosomal degradation alone (by NH_4Cl, chloroquine, or peptidase inhibitor E64) did not facilitate cross-presentation in MutuDC (Figure S6C), we also concluded that it is unlikely that prazosin acts primarily by protecting antigens from degradation. Together, these data indicate that facilitating antigen import into the cytosol overcomes the requirement for DC activation during cross-presentation and suggests that antigen import might be a key regulatory step that determines which antigens are destined for cross-presentation.

We went on to determine if endosomal processing of antigens for presentation on MHC class II was also enhanced by prazosin. We fed DCs with soluble endotoxin-free OVA, cell-associated OVA, or the appropriate peptides that bind directly to MHC molecules and co-cultured them with OT-I ($CD8^+$) and OT-II ($CD4^+$) T cells (specific for $K^b/OVA_{257-264}$ and $I\text{-}A^b/OVA_{323-339}$, respectively). As shown in Figure 6H, presentation of soluble and cell-associated antigens to $CD4^+$ OT-II cells was not affected by prazosin. This was in clear contrast with Poly(I:C), which strongly enhanced sOVA presentation to $CD4^+$ but not to $CD8^+$ T cells. Again, prazosin did not enhance T cell priming when DCs were treated with the short peptides directly, which would not require import into the cytosol for presentation. In summary, prazosin enhances antigen cross-presentation selectively and independently of DC maturation.

Finally, we investigated whether prazosin could be used to enhance antigen cross-presentation and anti-tumor immunity *in vivo*. In mice bearing MC38-GFP-OVA tumors, we observed reduced tumor growth following systemic treatment with prazosin (Figure 6I). In the more aggressive tumor model B16-OVA, prazosin alone was insufficient to control tumor growth and neither was a checkpoint inhibitor, anti-PD-1. However, combination of prazosin and anti-PD-1 led to a synergistic effect and a significant delay in tumor growth (Figure 6J). To rule out that this growth delay was caused by a direct effect of the treatment on the tumor, we implanted B16 tumors in immunodeficient non-obese diabetic (NOD)/severe combined immunodeficiency (SCID)/IL-2Rγ-null mice (NSG). In the NSG mice, we did not observe any reduction of tumor volume with PD-1/prazosin treatment (Figure S6D). We conclude that a combination of checkpoint blockade and increased antigen cross-presentation can overcome resistance of certain tumors to immunotherapy.

DISCUSSION

In this study, we developed a strategy to harness the natural capacity of DCs to cross-present antigens by modulating a specific step involved in antigen processing: import into the cytosol. To enhance antigen import, we used small molecules identified through a pharmacological screen. We demonstrated that import is a rate-limiting step for cross-presentation, in particular for antigens free of pathogen-derived signals. This observation reinforces the hypothesis that import into the cytosol is a regulated step (Zehner et al., 2015), and implies that endogenous signals that drive import and cross-presentation in the absence of infection await identification.

Boosting antigen import and cross-presentation synergizes with anti-PD-1-mediated immunotherapy in a tumor model unresponsive to the antibody alone, suggesting that cytosolic antigen cross-presentation plays a critical role in anti-tumor immune responses. Thus, enhancing antigen cross-presentation with small molecules provides a strategy for combination therapies with checkpoint blockers. This approach of enhancing anti-tumor immunity still relies on the presence of neoantigens. Yet, it has a major advantage over tumor antigen-containing vaccines, in that enhancing the natural capacity of DCs to route internalized antigens for cross-presentation does not require prior identification of specific epitopes.

We report here over 30 FDA-approved small molecule enhancers of antigen import, and we characterize the effects of two molecules (prazosin and tamoxifen) in more detail. Using a combination of proteomics, microscopy, and bioinformatics,

(C) Phagocytosis efficiency in the presence and absence of prazosin. 3T3s were labeled with PKH26, and acquisition of the dye by MutuDCs was analyzed after 2 h of co-culture. Mean from three independent experiments ± SE.

(D) MutuDCs were incubated with sOVA or sOVA EF for 45 min followed by 3.5 h incubation with prazosin. B3Z assay was used to monitor cross-presentation efficiency (representative plot from three independent experiments, error bars indicate SEM from technical duplicates).

(E) For the analysis of DC activation, MutuDCs were incubated with sOVA/EF sOVA in the presence and in the absence of prazosin for 5 h, washed, further incubated for 16 h at 37°C, and stained with anti-CD86 (gated for live cells only).

(F) MutuDCs were incubated with the MHC I peptide in the presence or absence of prazosin for 5 h, washed, fixed, and incubated with B3Z hybridoma for 16 h. Mean from three independent experiments ±SE.

(G) MutuDCs were incubated with sOVA EF in the presence of indicated compounds for 5 h, and antigen cross-presentation was detected with B3Z hybridomas. Mean from three independent experiments ±SE.

(H) The effect of prazosin on antigen presentation to OT-I and OT-II cells. MutuDC were incubated with sOVA EF, OVA-expressing 3T3s, or MHC class I or II peptides in the presence or absence of prazosin or Poly(I:C).

(I) Tumor growth. Mice were injected subcutaneously (SC) with the MC38-OVA tumor cell. When tumors became detectable, the animals were treated systemically (intraperitoneally, i.p.) with 0.5 mg prazosin or vehicle control, 3× week. Mice pooled from two independent experiments. The numbers indicate number of mice with tumors smaller than 250 mm^3 at the end of the experiment. Lower panel represents best-fit curves for control and prazosin-treated groups, where means and SD were calculated using loess regression, the statistical significance was calculated using ANOVA with the Tukey test and false discovery rate (FDR) Benjamini-Hochberg correction. ***$p < 0.001$ ****$p < 0.0001$.

(J) Tumor growth curves for mice injected s.c. with the B16-OVA tumor cells. From the day when tumors became detectable, mice were treated three times per week with 0.5 mg prazosin, 150 μg anti-PD-1, or the combination of both. Mice pooled from three independent experiments. The last panel represents best-fit curves for all groups, where means and SD were calculated using loess regression. The statistical significance was calculated using ANOVA with the Dunnett's test and FDR Benjamini-Hochberg correction. **$p < 0.01$, ****$p < 0.0001$.

See also Figure S6.

we concluded that import enhancement occurs as a consequence of lysosomal trapping of the drugs and increased lysosomal permeability. Other lysosomotropic compounds, such as chloroquine and NH_4Cl, have been shown to raise endolysosomal pH, inhibit lysosomal degradation, and as a result, increase cross-presentation efficiency (Accapezzato et al., 2005; Belizaire and Unanue, 2009; Chatterjee et al., 2012; Hotta et al., 2006; Ménager et al., 2014). Intriguingly, this effect was observed primarily in macrophages and human monocyte-derived DCs; in immature mouse bone-marrow-derived DCs, chloroquine and NH_4Cl had either an inhibitory or no effect on cross-presentation (Datta et al., 2003; Jancic et al., 2007; Kovacsovics-Bankowski and Rock, 1995; Oura et al., 2011; Rodriguez et al., 1999; Sehrawat et al., 2013). Neither NH_4Cl nor chloroquine increased the efficiency of antigen import or cross-presentation in MutuDCs, and instead, they abolished the effect of import enhancers. The observation that antigens internalized by cDC1s may persist for a longer time (Thacker and Janssen, 2012; Reboulet et al., 2010) could explain why inhibiting lysosomal degradation has also no effect on cross-presentation in MutuDCs. Therefore, accumulation in lysosomes is necessary for the activity of the small molecules we identified in this study, but lysosomotropism in itself does not promote endolysosomal permeability.

A number of mechanisms have been proposed by which lysosomotropic compounds could destabilize membranes. For instance, sunitinib and mefloquine, present among our top hits, have the ability to directly fluidize lysosomal membranes (Zhitomirsky et al., 2018). Other enhancers of antigen import, such as chlorpromazine, perphenazine, or fluphenazine displace lysosomal lipid hydrolases from the inner leaflet of the lysosome and destabilize membranes by inducing changes in their lipid composition (Kornhuber et al., 2010). Although the lysosomotropism of phenothiazines has been extensively studied, lysosomotropic properties (and corresponding effects) of quinazolinamine-derived compounds—the most potent chemical group in the antigen import assay—have not been studied in detail. Interestingly, the drug library used here also includes other compounds previously shown to permeabilize lysosomes that were not active in the β-lactamase assay (e.g., norfloxacin and ciprofloxacin, which destabilize lysosomes in cancer cells). Further work will be required to determine what confers specificity of the compounds, but differences in pH, membrane composition, and proteolytic content of endolysosomal compartments are likely to influence the extent and consequences of lysosomal trapping for the different classes of lysosomotropic compounds.

Many of the clinically approved drugs demonstrate unexpected activities that have either a harmful or a beneficial effect for the patient (Pushpakom et al., 2019). These hidden effects can also be exploited for new therapeutic indications in drug repositioning approaches. Yet, predicting or identifying effects of small molecules on target cells remains challenging. Here, we took advantage of the fact that localization and/or trafficking patterns of proteins are integral to most aspects of cellular functions. We demonstrated that comparative organellar mapping provides an effective and generic strategy for unbiased identification of biological effects of small molecules. This approach can be used to characterize changes in subcellular localization of thousands of proteins simultaneously (without the need for antibodies or protein tagging) and to characterize on- and off-target effects in any cell type of choice (including primary cells). To our knowledge, this is a first report of organellar mapping in cDC1s (or dendritic cells altogether), and it provides a useful resource of information on subcellular localization and abundance of poorly characterized proteins that are not expressed in common cell lines (organellar map and full proteome composition are available at http://dc-biology.mrc-lmb.cam.ac.uk).

In summary, through a combination of small molecule screening and proteomics-based molecular mapping, we established an approach for enhancing presentation of antigens sampled by DCs in the absence of strong immunogenic signals. Enhancing cross-presentation with small molecules may in the future provide therapeutic regimes for patients that do not respond to currently available treatment options.

STAR★METHODS

Detailed methods are provided in the online version of this paper and include the following:

- KEY RESOURCES TABLE
- RESOURCE AVAILABILITY
  - Lead Contact
  - Materials Availability
  - Data and Code Availability
- EXPERIMENTAL MODELS
  - Animals
  - Cell lines and cell culture
- METHOD DETAILS
  - Compounds and antibodies
  - Cell culture
  - Antigen import assay and library screen
  - Chemical class and target assignment
  - Cross-presentation assay
  - OTI and OTII activation assays
  - Cross-presentation of cell-associated antigens
  - Cell uptake assay
  - DC activation
  - Live microscopy
  - Galectin 3-YFP accumulation
  - Dextran release assays
  - Prazosin-BODIPY accumulation
  - MC38-GFP-OVA tumour growth experiments
  - B16-OVA tumour growth experiments
  - Whole cell proteomics
  - Proteomic analysis of cytosol
  - Dynamic organellar maps
  - Mass spectrometry and data processing
  - Bioinformatic analysis of the proteomic data
  - Copy number estimates of proteins expressed in MutuDC
  - Drug-induced changes in whole cell proteomes
  - Drug-induced changes in cytosol
  - Organellar maps
  - Subcellular localization predictions in MutuDC

- ○ Drug induced protein movements
- QUANTIFICATION AND STATISTICAL ANALYSIS
- ADDITIONAL RESOURCES

SUPPLEMENTAL INFORMATION

Supplemental Information can be found online at https://doi.org/10.1016/j.celrep.2020.107905.

ACKNOWLEDGMENTS

We would like to thank Hans Acha-Orbea for the MutuDCs, Matthew Albert for the NIH/3T3 cells expressing a non-secretable form of OVA, Felix Randow for the galectin-3-YFP construct, Jon Howe for help with microscopy, and Greg Słodkowicz for help with statistical analysis. D.N.I. and G.H.H.B. also wish to thank Matthias Mann for his support. S.A. received funding from Institute Curie; Institut National de la Santé et de la Recherche Médicale; Centre National de la Recherche Scientifique; la Ligue Contre le Cancer (Equipe labellisée Ligue, EL2014.LNCC/SA); Association de Recherche Contre le Cancer (ARC); the ERC (2013-AdG 340046 DCBIOX); INCA (PLBIO13-057); ANR-11-LABX-0043 and ANR-10-IDEX-0001-02 PSL; and ANR-16-CE15001801 and ANR-16-CE18002003. P.K. was supported by EMBO (ALTF 467-2012), the Wellcome Trust (101578/Z/13/Z), and Medical Research Council (U105178805). A.A. was supported by EMBO (ALTF 883-2011). D.N.I. was funded by the Louis-Jeantet Foundation and the Max Planck Society for the Advancement of Science. G.H.H.B. was funded by the German Research Foundation (DFG/Gottfried Wilhelm Leibniz Prize) and the Max Planck Society for the Advancement of Science.

AUTHOR CONTRIBUTIONS

P.K. performed, analyzed, designed, and supervised most of the experiments. M.G. performed and analyzed part of the antigen presentation assays and set up and carried out experiments with cell-associated antigens. D.N.I. carried out the proteomic experiments and assisted with data analysis. L.J. and S.H.-C. performed tumor experiments and assisted with animal work. P.A.K. assisted with the B3Z assays. P.R.-S. assisted with the dextran release assays. M.G. and A.A. assisted with tumor experiments. J.G.M. assisted in assay development and experimental design. E.D.N. assisted with design of the small molecule screening assays. G.H.H.B. designed the proteomics experiments, performed the data analysis, and supervised the proteomics work. P.K. and S.A. conceived and supervised the study. P.K., G.H.H.B., and S.A. wrote the manuscript.

DECLARATION OF INTERESTS

The authors declare no competing interests.

Received: September 16, 2019
Revised: May 15, 2020
Accepted: June 24, 2020
Published: July 14, 2020

REFERENCES

Accapezzato, D., Visco, V., Francavilla, V., Molette, C., Donato, T., Paroli, M., Mondelli, M.U., Doria, M., Torrisi, M.R., and Barnaba, V. (2005). Chloroquine enhances human CD8+ T cell responses against soluble antigens in vivo. J. Exp. Med. *202*, 817–828.

Ackerman, A.L., Kyritsis, C., Tampé, R., and Cresswell, P. (2003). Early phagosomes in dendritic cells form a cellular compartment sufficient for cross presentation of exogenous antigens. Proc. Natl. Acad. Sci. USA *100*, 12889–12894.

Alloatti, A., Kotsias, F., Magalhaes, J.G., and Amigorena, S. (2016). Dendritic cell maturation and cross-presentation: timing matters!. Immunol. Rev. *272*, 97–108.

Alloatti, A., Rookhuizen, D.C., Joannas, L., Carpier, J.-M., Iborra, S., Magalhaes, J.G., Yatim, N., Kozik, P., Sancho, D., Albert, M.L., and Amigorena, S. (2017). Critical role for Sec22b-dependent antigen cross-presentation in antitumor immunity. J. Exp. Med. *214*, 2231–2241.

Belizaire, R., and Unanue, E.R. (2009). Targeting proteins to distinct subcellular compartments reveals unique requirements for MHC class I and II presentation. Proc. Natl. Acad. Sci. USA *106*, 17463–17468.

Bonifaz, L., Bonnyay, D., Mahnke, K., Rivera, M., Nussenzweig, M.C., and Steinman, R.M. (2002). Efficient targeting of protein antigen to the dendritic cell receptor DEC-205 in the steady state leads to antigen presentation on major histocompatibility complex class I products and peripheral CD8+ T cell tolerance. J. Exp. Med. *196*, 1627–1638.

Borner, G.H.H. (2020). Organellar maps through proteomic profiling - a conceptual guide. Mol. Cell. Proteomics *19*, 1076–1087.

Böttcher, J.P., and Reis e Sousa, C. (2018). The Role of Type 1 Conventional Dendritic Cells in Cancer Immunity. Trends Cancer *4*, 784–792.

Brunner, C., Seiderer, J., Schlamp, A., Bidlingmaier, M., Eigler, A., Haimerl, W., Lehr, H.A., Krieg, A.M., Hartmann, G., and Endres, S. (2000). Enhanced dendritic cell maturation by TNF-alpha or cytidine-phosphate-guanosine DNA drives T cell activation in vitro and therapeutic anti-tumor immune responses in vivo. J. Immunol. *165*, 6278–6286.

Burgdorf, S., Schölz, C., Kautz, A., Tampé, R., and Kurts, C. (2008). Spatial and mechanistic separation of cross-presentation and endogenous antigen presentation. Nat. Immunol. *9*, 558–566.

Caminschi, I., Proietto, A.I., Ahmet, F., Kitsoulis, S., Shin Teh, J., Lo, J.C.Y., Rizzitelli, A., Wu, L., Vremec, D., van Dommelen, S.L.H., et al. (2008). The dendritic cell subtype-restricted C-type lectin Clec9A is a target for vaccine enhancement. Blood *112*, 3264–3273.

Cebrian, I., Visentin, G., Blanchard, N., Jouve, M., Bobard, A., Moita, C., Enninga, J., Moita, L.F., Amigorena, S., and Savina, A. (2011). Sec22b regulates phagosomal maturation and antigen crosspresentation by dendritic cells. Cell *147*, 1355–1368.

Chatterjee, B., Smed-Sörensen, A., Cohn, L., Chalouni, C., Vandlen, R., Lee, B.-C., Widger, J., Keler, T., Delamarre, L., and Mellman, I. (2012). Internalization and endosomal degradation of receptor-bound antigens regulate the efficiency of cross presentation by human dendritic cells. Blood *120*, 2011–2020.

Cox, J., and Mann, M. (2008). MaxQuant enables high peptide identification rates, individualized p.p.b.-range mass accuracies and proteome-wide protein quantification. Nat. Biotechnol. *26*, 1367–1372.

Crittenden, M.R., Zebertavage, L., Kramer, G., Bambina, S., Friedman, D., Troesch, V., Blair, T., Baird, J.R., Alice, A., and Gough, M.J. (2018). Tumor cure by radiation therapy and checkpoint inhibitors depends on pre-existing immunity. Sci. Rep. *8*, 7012.

Datta, S.K., Redecke, V., Prilliman, K.R., Takabayashi, K., Corr, M., Tallant, T., DiDonato, J., Dziarski, R., Akira, S., Schoenberger, S.P., and Raz, E. (2003). A subset of Toll-like receptor ligands induces cross-presentation by bone marrow-derived dendritic cells. J. Immunol. *170*, 4102–4110.

Falo, L.D., Jr., Kovacsovics-Bankowski, M., Thompson, K., and Rock, K.L. (1995). Targeting antigen into the phagocytic pathway in vivo induces protective tumour immunity. Nat. Med. *1*, 649–653.

Fuertes Marraco, S.A., Grosjean, F., Duval, A., Rosa, M., Lavanchy, C., Ashok, D., Haller, S., Otten, L.A., Steiner, Q.-G., Descombes, P., et al. (2012). Novel murine dendritic cell lines: a powerful auxiliary tool for dendritic cell research. Front. Immunol. *3*, 331.

Gilfillan, S., Chan, C.J., Cella, M., Haynes, N.M., Rapaport, A.S., Boles, K.S., Andrews, D.M., Smyth, M.J., and Colonna, M. (2008). DNAM-1 promotes activation of cytotoxic lymphocytes by nonprofessional antigen-presenting cells and tumors. J. Exp. Med. *205*, 2965–2973.

Giodini, A., and Cresswell, P. (2008). Hsp90-mediated cytosolic refolding of exogenous proteins internalized by dendritic cells. EMBO J. *27*, 201–211.

Gros, M., and Amigorena, S. (2019). Regulation of Antigen Export to the Cytosol During Cross-Presentation. Front. Immunol. *10*, 41.

Grotzke, J.E., Kozik, P., Morel, J.-D., Impens, F., Pietrosemoli, N., Cresswell, P., Amigorena, S., and Demangel, C. (2017). Sec61 blockade by mycolactone inhibits antigen cross-presentation independently of endosome-to-cytosol export. Proc. Natl. Acad. Sci. USA *114*, E5910–E5919.

Guermonprez, P., Saveanu, L., Kleijmeer, M., Davoust, J., Van Endert, P., and Amigorena, S. (2003). ER-phagosome fusion defines an MHC class I cross-presentation compartment in dendritic cells. Nature *425*, 397–402.

Hammerich, L., Marron, T.U., Upadhyay, R., Svensson-Arvelund, J., Dhainaut, M., Hussein, S., Zhan, Y., Ostrowski, D., Yellin, M., Marsh, H., et al. (2019). Systemic clinical tumor regressions and potentiation of PD1 blockade with in situ vaccination. Nat. Med. *25*, 814–824.

Hildner, K., Edelson, B.T., Purtha, W.E., Diamond, M., Matsushita, H., Kohyama, M., Calderon, B., Schraml, B.U., Unanue, E.R., Diamond, M.S., et al. (2008). Batf3 deficiency reveals a critical role for CD8α+ dendritic cells in cytotoxic T cell immunity. Science *322*, 1097–1100.

Hotta, C., Fujimaki, H., Yoshinari, M., Nakazawa, M., and Minami, M. (2006). The delivery of an antigen from the endocytic compartment into the cytosol for cross-presentation is restricted to early immature dendritic cells. Immunology *117*, 97–107.

Imai, J., Hasegawa, H., Maruya, M., Koyasu, S., and Yahara, I. (2005). Exogenous antigens are processed through the endoplasmic reticulum-associated degradation (ERAD) in cross-presentation by dendritic cells. Int. Immunol. *17*, 45–53.

Itzhak, D.N., Tyanova, S., Cox, J., and Borner, G.H. (2016). Global, quantitative and dynamic mapping of protein subcellular localization. eLife *5*, e16950.

Itzhak, D.N., Davies, C., Tyanova, S., Mishra, A., Williamson, J., Antrobus, R., Cox, J., Weekes, M.P., and Borner, G.H.H. (2017). A Mass Spectrometry-Based Approach for Mapping Protein Subcellular Localization Reveals the Spatial Proteome of Mouse Primary Neurons. Cell Rep. *20*, 2706–2718.

Itzhak, D.N., Schessner, J.P., and Borner, G.H.H. (2019). Dynamic Organellar Maps for Spatial Proteomics. Curr. Protoc. Cell Biol. *83*, e81–e29.

Jancic, C., Savina, A., Wasmeier, C., Tolmachova, T., El-Benna, J., Dang, P.M.-C., Pascolo, S., Gougerot-Pocidalo, M.-A., Raposo, G., Seabra, M.C., and Amigorena, S. (2007). Rab27a regulates phagosomal pH and NADPH oxidase recruitment to dendritic cell phagosomes. Nat. Cell Biol. *9*, 367–378.

Kornhuber, J., Tripal, P., Reichel, M., Mühle, C., Rhein, C., Muehlbacher, M., Groemer, T.W., and Gulbins, E. (2010). Functional Inhibitors of Acid Sphingomyelinase (FIASMAs): a novel pharmacological group of drugs with broad clinical applications. Cell. Physiol. Biochem. *26*, 9–20.

Kovacsovics-Bankowski, M., and Rock, K.L. (1995). A phagosome-to-cytosol pathway for exogenous antigens presented on MHC class I molecules. Science *267*, 243–246.

Kulak, N.A., Pichler, G., Paron, I., Nagaraj, N., and Mann, M. (2014). Minimal, encapsulated proteomic-sample processing applied to copy-number estimation in eukaryotic cells. Nat. Methods *11*, 319–324.

Law, V., Knox, C., Djoumbou, Y., Jewison, T., Guo, A.C., Liu, Y., Maciejewski, A., Arndt, D., Wilson, M., Neveu, V., et al. (2014). DrugBank 4.0: shedding new light on drug metabolism. Nucleic Acids Res. *42*, D1091–D1097.

Lundberg, E., and Borner, G.H.H. (2019). Spatial proteomics: a powerful discovery tool for cell biology. Nat. Rev. Mol. Cell Biol. *20*, 285–302.

MacDonald, M.L., Lamerdin, J., Owens, S., Keon, B.H., Bilter, G.K., Shang, Z., Huang, Z., Yu, H., Dias, J., Minami, T., et al. (2006). Identifying off-target effects and hidden phenotypes of drugs in human cells. Nat. Chem. Biol. *2*, 329–337.

Ménager, J., Ebstein, F., Oger, R., Hulin, P., Nedellec, S., Duverger, E., Lehmann, A., Kloetzel, P.-M., Jotereau, F., and Guilloux, Y. (2014). Cross-presentation of synthetic long peptides by human dendritic cells: a process dependent on ERAD component p97/VCP but Not sec61 and/or Derlin-1. PLoS ONE *9*, e89897.

Nadanaciva, S., Lu, S., Gebhard, D.F., Jessen, B.A., Pennie, W.D., and Will, Y. (2011). A high content screening assay for identifying lysosomotropic compounds. Toxicol. In Vitro *25*, 715–723.

Ott, P.A., Hu, Z., Keskin, D.B., Shukla, S.A., Sun, J., Bozym, D.J., Zhang, W., Luoma, A., Giobbie-Hurder, A., Peter, L., et al. (2017). An immunogenic personal neoantigen vaccine for patients with melanoma. Nature *547*, 217–221.

Oura, J., Tamura, Y., Kamiguchi, K., Kutomi, G., Sahara, H., Torigoe, T., Himi, T., and Sato, N. (2011). Extracellular heat shock protein 90 plays a role in translocating chaperoned antigen from endosome to proteasome for generating antigenic peptide to be cross-presented by dendritic cells. Int. Immunol. *23*, 223–237.

Palmowski, M.J., Gileadi, U., Salio, M., Gallimore, A., Millrain, M., James, E., Addey, C., Scott, D., Dyson, J., Simpson, E., and Cerundolo, V. (2006). Role of immunoproteasomes in cross-presentation. J. Immunol. *177*, 983–990.

Pushpakom, S., Iorio, F., Eyers, P.A., Escott, K.J., Hopper, S., Wells, A., Doig, A., Guilliams, T., Latimer, J., McNamee, C., et al. (2019). Drug repurposing: progress, challenges and recommendations. Nat. Rev. Drug Discov. *18*, 41–58.

Reboulet, R.A., Hennies, C.M., Garcia, Z., Nierkens, S., and Janssen, E.M. (2010). Prolonged antigen storage endows merocytic dendritic cells with enhanced capacity to prime anti-tumor responses in tumor-bearing mice. J. Immunol. *185*, 3337–3347.

Rock, K.L., and Shen, L. (2005). Cross-presentation: underlying mechanisms and role in immune surveillance. Immunol. Rev. *207*, 166–183.

Rodriguez, A., Regnault, A., Kleijmeer, M., Ricciardi-Castagnoli, P., and Amigorena, S. (1999). Selective transport of internalized antigens to the cytosol for MHC class I presentation in dendritic cells. Nat. Cell Biol. *1*, 362–368.

Salmon, H., Idoyaga, J., Rahman, A., Leboeuf, M., Remark, R., Jordan, S., Casanova-Acebes, M., Khudoynazarova, M., Agudo, J., Tung, N., et al. (2016). Expansion and Activation of CD103(+) Dendritic Cell Progenitors at the Tumor Site Enhances Tumor Responses to Therapeutic PD-L1 and BRAF Inhibition. Immunity *44*, 924–938.

Sánchez-Paulete, A.R., Cueto, F.J., Martínez-López, M., Labiano, S., Morales-Kastresana, A., Rodríguez-Ruiz, M.E., Jure-Kunkel, M., Azpilikueta, A., Aznar, M.A., Quetglas, J.I., et al. (2016). Cancer Immunotherapy with Immunomodulatory Anti-CD137 and Anti-PD-1 Monoclonal Antibodies Requires BATF3-Dependent Dendritic Cells. Cancer Discov. *6*, 71–79.

Sánchez-Paulete, A.R., Teijeira, Á., Quetglas, J.I., Rodríguez-Ruiz, M.E., Sánchez-Arráez, Á., Labiano, S., Etxeberria, I., Azpilikueta, A., Bolaños, E., Ballesteros-Briones, M.C., et al. (2018). Intratumoral Immunotherapy with XCL1 and sFlt3L Encoded in Recombinant Semliki Forest Virus-Derived Vectors Fosters Dendritic Cell-Mediated T-cell Cross-Priming. Cancer Res. *78*, 6643–6654.

Sancho, D., Mourão-Sá, D., Joffre, O.P., Schulz, O., Rogers, N.C., Pennington, D.J., Carlyle, J.R., and Reis e Sousa, C. (2008). Tumor therapy in mice via antigen targeting to a novel, DC-restricted C-type lectin. J. Clin. Invest. *118*, 2098–2110.

Sanderson, S., and Shastri, N. (1994). LacZ inducible, antigen/MHC-specific T cell hybrids. Int. Immunol. *6*, 369–376.

Schindelin, J., Arganda-Carreras, I., Frise, E., Kaynig, V., Longair, M., Pietzsch, T., Preibisch, S., Rueden, C., Saalfeld, S., Schmid, B., et al. (2012). Fiji: an open-source platform for biological-image analysis. Nat. Methods *9*, 676–682.

Sehrawat, S., Koenig, P.-A., Kirak, O., Schlieker, C., Fankhauser, M., and Ploegh, H.L. (2013). A catalytically inactive mutant of the deubiquitylase YOD-1 enhances antigen cross-presentation. Blood *121*, 1145–1156.

Shen, L., Sigal, L.J., Boes, M., and Rock, K.L. (2004). Important role of cathepsin S in generating peptides for TAP-independent MHC class I cross-presentation in vivo. Immunity *21*, 155–165.

Thacker, R.I., and Janssen, E.M. (2012). Cross-presentation of cell-associated antigens by mouse splenic dendritic cell populations. Front. Immunol. *3*, 41.

Theisen, D.J., Davidson, J.T., 4th, Briseño, C.G., Gargaro, M., Lauron, E.J., Wang, Q., Desai, P., Durai, V., Bagadia, P., Brickner, J.R., et al. (2018). WDFY4 is required for cross-presentation in response to viral and tumor antigens. Science *362*, 694–699.

Thurston, T.L.M., Wandel, M.P., von Muhlinen, N., Foeglein, A., and Randow, F. (2012). Galectin 8 targets damaged vesicles for autophagy to defend cells against bacterial invasion. Nature *482*, 414–418.

CellPress
OPEN ACCESS

Tyanova, S., Temu, T., Sinitcyn, P., Carlson, A., Hein, M.Y., Geiger, T., Mann, M., and Cox, J. (2016). The Perseus computational platform for comprehensive analysis of (prote)omics data. Nat. Methods *13*, 731–740.

Wickham, H., Averick, M., Bryan, J., Chang, W., McGowan, L., François, R., Grolemund, G., Hayes, A., Henry, L., Hester, J., et al. (2019). Welcome to the Tidyverse. J. Open Source Softw. *4*, 1686.

Wiśniewski, J.R., Zougman, A., Nagaraj, N., and Mann, M. (2009). Universal sample preparation method for proteome analysis. Nat. Methods *6*, 359–362.

Wiśniewski, J.R., Hein, M.Y., Cox, J., and Mann, M. (2014). A "proteomic ruler" for protein copy number and concentration estimation without spike-in standards. Mol. Cell. Proteomics *13*, 3497–3506.

Yoshida, H., Lareau, C.A., Ramirez, R.N., Rose, S.A., Maier, B., Wroblewska, A., Desland, F., Chudnovskiy, A., Mortha, A., Dominguez, C., et al.; Immunological Genome Project (2019). The cis-Regulatory Atlas of the Mouse Immune System. Cell *176*, 897–912.

Zehner, M., Marschall, A.L., Bos, E., Schloetel, J.-G., Kreer, C., Fehrenschild, D., Limmer, A., Ossendorp, F., Lang, T., Koster, A.J., et al. (2015). The translocon protein Sec61 mediates antigen transport from endosomes in the cytosol for cross-presentation to CD8(+) T cells. Immunity *42*, 850–863.

Zhitomirsky, B., Yunaev, A., Kreiserman, R., Kaplan, A., Stark, M., and Assaraf, Y.G. (2018). Lysosomotropic drugs activate TFEB via lysosomal membrane fluidization and consequent inhibition of mTORC1 activity. Cell Death Dis. *9*, 1191.

Zlokarnik, G., Negulescu, P.A., Knapp, T.E., Mere, L., Burres, N., Feng, L., Whitney, M., Roemer, K., and Tsien, R.Y. (1998). Quantitation of transcription and clonal selection of single living cells with beta-lactamase as reporter. Science *279*, 84–88.

STAR★METHODS

KEY RESOURCES TABLE

REAGENT or RESOURCE	SOURCE	IDENTIFIER
Antibodies		
anti-CD11c-FITC (clone HL3)	BD PharMingen	Cat#553801; RRID:AB_395060
anti-CD19-eFluor®450 (clone 1D3)	eBioscience	Cat#48-0193-82; RRID:AB_2734905
anti-CD25-FITC (clone 7D4)	BD PharMingen	Cat#553072; RRID:AB_394604
anti-CD25-PerCPCy5.5 (clone PC61)	BD PharMingen	Cat#551071; RRID:AB_394031
anti-CD3-eFluor®450 (clone 17A2)	eBioscience	Cat#48-0032-80; RRID:AB_1272229
anti-CD4-APC (clone RM4-5)	BD PharMingen	Cat#553051; RRID:AB_398528
anti-CD4-PE-Cy7 (clone RM4-5)	BD PharMingen	Cat#552775; RRID:AB_394461
anti-CD69-eFluor®450 (clone H1.2F3)	eBioscience	Cat#48-0691-82; RRID:AB_10719430
anti-CD69-PE (clone H1.2F3)	BD PharMingen	Cat#553237; RRID:AB_394726
anti-CD86-PE (clone GL1)	BD PharMingen	Cat#553692; RRID:AB_394994
anti-CD8a-PerCP-Cy5.5 (clone 53-6.7)	eBioscience	Cat#45-0081-82; RRID:AB_1107004
anti-CD8α-Pacific Blue (clone 53-6.7)	BD PharMingen	Cat#558106; RRID:AB_397029
anti-CD8α-PeCy7 (clone 53-6.7)	BD PharMingen	Cat#552877; RRID:AB_394506
anti-MHC I (H-2Kb)-FITC (clone AF6-88.5.5.3)	eBioscience	Cat#11-5958-80; RRID:AB_11151335
anti-MHC II eFluor®450 (clone AF120.1, eBioscience)	eBioscience	Cat#48-5320-80; RRID:AB_10671538
anti-vβ 5.1 5.2 TCR-PE (clone MR9-4)	BD PharMingen	Cat#553190; RRID:AB_394698
anti-Vα2 TCR-eFluor®450 (clone B20.1)	eBioscience	Cat#48-5812-82; RRID:AB_10804752
anti-Vα2 TCR-PeCy7 (clone B20.1)	BD PharMingen	Cat#560624; RRID:AB_1727584
Chemicals, Peptides, and Recombinant Proteins		
β-lactamase	Sigma-Aldrich	Cat#P0389
BODIPY	ThermoFisher	Cat#D-3922
Chloroquine	Sigma-Aldrich	Cat#C6628
DbeQ	Sigma-Aldrich	Cat#SML0031
Dextran, 3000 MW Tetramethylrhodamine-labeled	ThermoFisher	Cat#D3307
E64	Sigma-Aldrich	Cat#E3132
Eeyarestatin I	Sigma-Aldrich	Cat#E1286
Importazole	Sigma-Aldrich	Cat#SML0341
NMS-873	Selleckcheck	Cat#S7285
Ovalbumin (endotoxin-free)	Hyglos	Cat#300036
Ovalbumin (endotoxin-free)	Invivogen	Cat#vac-pova
Ovalbumin (grade VII)	Sigma Aldrich	Cat#A7641
PR-619	Sigma-Aldrich	Cat#SML0430
Prazosin (*in vitro* experiments)	Sigma-Aldrich	Cat#P7791
Prazosin (*in vivo* experiments)	Sigma-Aldrich	Cat#1554705
Prazosin-BODIPY	ThermoFisher	Cat#B7433
SCREEN-WELL® FDA approved drug library V2	Enzo	Cat#BML-2843-0100
Tamoxifen	Sigma-Aldrich	Cat#T9262
β-lactamase	Sigma-Aldrich	Cat#P0389
Critical Commercial Assays		
CCF2-FA	ThermoFisher	Cat#K1034
Fixable Viability Dye eFluor® 780	eBioscience	Cat#65-0865-14
LiveBLAzer FRET-B/G Loading Kit	ThermoFisher	Cat#K1095

(*Continued on next page*)

Continued

REAGENT or RESOURCE	SOURCE	IDENTIFIER
Deposited Data		
Proteomics data: whole cell proteomics of MutuDCs, organellar mapping of MutuDCs	This paper	http://dc-biology.mrc-lmb.cam.ac.uk
Experimental Models: Cell Lines		
B16-OVA	Falo et al., 1995	RRID:CVCL_WM78
B3Z	Sanderson and Shastri, 1994	RRID:CVCL_6277
MC38-OVA	Gilfillan et al., 2008	N/A
MutuDC	Fuertes Marraco et al., 2012	N/A
Experimental Models: Organisms/Strains		
Mouse: C57BL/6J (wild type)	Charles River	Cat#632
Mouse: C57BL/6-Rag1^{tm1Mom}Tg(TcraTcrb)1100Mjb	Taconic	Cat#4175
Mouse: C57BL/6-Tg(TcraTcrb)1100Mjb/Crl (OT-1)	Charles River	Cat#642
Mouse: C57BL/6-Tg(TcraTcrb)425Cbn/Crl (OT-2)	Charles River	Cat#643
Mouse: NOD.Cg-Prkdc^{scid} Il2rg^{tm1Wjl}/SzJ (NSG^{TM})	Jackson Laboratory	Cat#005557
Software and Algorithms		
ImageJ/FIJI	Schindelin et al., 2012	https://imagej.nih.gov/ij/
GraphPad Prism version 8.4.2	GraphPad Software, La Jolla California USA	https://www.graphpad.com
MaxQuant version 1.6	Cox and Mann, 2008	https://www.maxquant.org/
Perseus version 1.6	Tyanova et al., 2016	https://www.maxquant.org/
R version 3.5.3	The R Foundation	https://www.R-project.org/
Tidyverse version 1.3.0	Wickham et al., 2019	https://www.tidyverse.org/

RESOURCE AVAILABILITY

Lead Contact

Further information and requests for resources and reagents should be directed to and will be fulfilled by the Lead Contact, Patrycja Kozik (pkozik@mrc-lmb.cam.ac.uk).

Materials Availability

This study did not generate unique reagents.

Data and Code Availability

The datasets generated during this study are provided as Supplemental Information and as a web resource at http://dc-biology.mrc-lmb.cam.ac.uk.

EXPERIMENTAL MODELS

Animals

C57BL/6J wild-type mice, OT-I, Rag1-deficient OT-I, and OT-II transgenic mice were obtained from Charles River Laboratories, Janvier and Centre de Distribution, Typage et Archive Animal (CDTA, Orleans, France). NOD.Cg-Prkdc^{scid}Il2rg^{tm1Wjl}/SzJ (NOD scid gamma, NSG^{TM}) mice were originally purchased from the Jackson Laboratory and bred in our animal facility under specific pathogen-free conditions. Mice were used between 8-12 weeks old and were gender matched within each experiment (both genders were used).

All animal procedures were in accordance with the guidelines and regulations of the Institut Curie Veterinary Department and all mice used were less than six months old.

Cell lines and cell culture

The following cell lines were used in this study: GFP^{+} MutuDC, obtained from Hans-Acha Orbea (Fuertes Marraco et al., 2012), NIH/3T3 expressing a non-secretable form of OVA obtained from Matthew Albert, B3Z hybridoma cells (Sanderson and Shastri, 1994), B16-OVA cells (Falo et al., 1995), MC38-OVA (Gilfillan et al., 2008).

All cell lines testes as mycoplasma-negative by PCR.

METHOD DETAILS

Compounds and antibodies

For flow cytometry, the following antibodies were used: anti-CD86-PE (clone GL1, BD PharMingen Cat#553692), anti-CD69-PE (clone H1.2F3, BD PharMingen Cat#553237), anti-CD25-PerCPCy5.5 (clone PC61, BD PharMingen Cat#551071), anti-CD4-APC (clone RM4-5, BD PharMingen Cat#553051), anti-CD8α-Pacific Blue (clone 53-6.7, BD PharMingen Cat#558106), anti-Vα2-PeCy7 (clone B20.1, BD PharMingen Cat#560624), anti-CD8α-PeCy7 (clone 53-6.7, BD PharMingen Cat#552877), anti-Vα2-eFluor®450 (clone B20.1, eBiosciences Cat#48-5812-82), anti-CD69-eFluor®450 (clone H1.2F3, eBiosciences Cat#48-0691-82), anti-CD25-FITC (clone 7D4, BD PharMingen Cat#553072), anti-CD8a-PerCP-Cy5.5 (clone 53-6.7, eBioscience, Cat#45-0081-82), anti-TCR vβ 5.1-PE (clone MR9-4, BD PharMingen Cat#553190), anti-CD4-PE-Cy7 (clone RM4-5, BD PharMingen Cat#552775), anti-CD19-eFluor®450 (clone 1D3, eBioscience, Cat#48-0193), anti-CD3-eFluor®450 (clone 17A2, eBioscience, Cat#48-0032-80), anti-CD11c-FITC (clone HL3, BD PharMingen Cat#553801), anti-MHC I (H-2Kb)-FITC (clone AF6-88.5.5.3, eBioscience Cat#11-5958-80), anti-MHC II-eFluor®450 (clone AF120.1, eBioscience Cat#48-5320-80).

The following small molecules were used (at the indicated concentrations, unless otherwise stated in the text): DbeQ (4 μM, Cat#SML0031), importazole (30 μM, Cat#SML0341), PR-619 (20 μM, Cat#SML0430), Eeyarestatin I (10 μM, Cat#E1286), prazosin (10 μM, Cat#P7791), prazosin (*in vivo* experiments, Cat#1554705) tamoxifen (10 μM, Cat#T9262), chloroquine (50 μM, Cat#C6628), E64 (2 μM Cat#E3132, all purchased from Sigma-Aldrich; Prazosin-BODIPY (5 μM, ThermoFisher Scientific, Cat#B7433), NMS-873 (10 μM, Selleckcheck, Cat#S7285), BODIPY (10 μg/ml, ThermoFisher Scientific, D-3922), SCREEN-WELL® FDA approved drug library V2 (Enzo, Cat#BML-2843-0100), CCF2-FA (ThermoFisher, Cat#K1034).

Cell culture

MutuDC were grown in IMDM, supplemented with 8% heat-inactivated FCS (Biowest-Biosera), 10 mM HEPES, 2 mM Glutamax, 100 IU/ml penicillin, 100 μg/ml streptomycin and 50 μM β-mercaptoethanol (all from Life Technologies).

For SILAC metabolic labeling, MutuDCs were grown in IMDM SILAC culture medium (Thermo, Cat#88423), supplemented with 8% (V/V) dialysed fetal calf serum (PAA, Cat#A11-107), 50 μM β-mercaptoethanol (GIBCO), Pencilin and Streptomycin (Sigma), 10 mM HEPES (pH 7.4), and either: 42 mg/L 13C6,15N4-L-Arginine HCl (Silantes, Cat#201604302) and 73 mg/L 13C6,15N2-L-Lysine HCl (Silantes, Cat#211604302) for SILAC heavy culture medium; or 42 mg/L L-Arginine HCl and 73 mg/L L-Lysine HCl with standard isotopic constituents (Sigma, Cat#A6969 and Cat#L8662) for SILAC light culture medium. Cells were allowed at least seven doublings prior to experiments, to ensure complete labeling.

NIH/3T3 expressing a non-secretable form of OVA were cultured in DMEM (Life Technologies) supplemented with 10% heat-inactivated FBS (Biowest-Biosera), 0.1 mM non-essential amino acids, 1 mM sodium pyruvate, 10 mM HEPES and 50 μM β-mercaptoethanol. Necroptosis was induced by treatment with a specific drug ligand (AP20187, BB homodimerizer, Clontech).

B3Z hybridoma cells were cultured in RPMI (Life Technologies), supplemented with 10% FBS (Biowest-Biosera), 0.1 mM non-essential amino acids, 1 mM sodium pyruvate, 10 mM HEPES and 50 μM β-mercaptoethanol, 10 mM HEPES.

B16-OVA cells were cultured in RPMI, supplemented with 10% heat-inactivated FCS (Biowest-Biosera), 2 mM Glutamax, 100 IU/ml penicillin and 100 μg/ml streptomycin (all from Life Technologies) and selected with G418 2 mg/ml (Life Technnologies) and hygromycin B 60 μg/ml (GIBCO).

MC38-OVA cells were grown in DMEM, supplemented with 10% heat-inactivated FCS (Biowest-Biosera), 2 mM Glutamax, 100 IU/ml penicillin and 100 μg/ml streptomycin (all from Life Technologies).

OT-I and OT-II T cells were isolated using EasySep Mouse Naive CD8+ and $CD4^+$ T Cell Isolation Kits respectively (Stemcell, Cat#19858 and Cat#19765) and cultured in the same media as the B3Z cells.

Antigen import assay and library screen

MutuDCs were seeded at 150,000 cells/well in U-bottom 96-well plates and incubated with 10 mg/ml β-lactamase (Sigma-Aldrich, Cat#P0389) for 3 h at 37°C. The cells were then washed and incubated with small molecules at indicated concentrations for 2 h at 37°C. CCF4 loading was performed using LiveBLAzer FRET-B/G Loading Kit (ThermoFisher, Cat#K1095) as described (Cebrian et al., 2011) for 45-60 min at RT. To increase the sensitivity of the assay, the plates were then incubated for 16 h at RT (Zlokarnik et al., 1998) in CO_2 independent media supplemented with 8% FCS, and 2 mM Glutamax (in the presence of compounds). Immediately before the flow cytometry analysis, the cells were stained with Fixable Viability Dye eFluor® 780 (eBioscience) diluted 1:2500 in PBS. Proportion of the live cells with a high ratio of blue to green (V450/V530) fluorescence was used as a measure of the efficiency of antigen import into the cytosol.

Primary screen of the FDA library. Enzo FDA-approved drug library was screened in the course of three independent experiments. Each 96-well plate contained three media-only, DMSO only, 4 μM DbeQ (enhancement control), and 10 μM PR-619 (inhibition control) wells to control for data reproducibility between the plates. The screen was performed once and 37 top ranked compounds were selected for validation.

Validation screen. The secondary screen was performed at six concentrations (1.25 – 40 μM) for each compound, in two biological repeats. Media-only and vehicle (DMSO) controls were included on each plate. Wells with less than 500 cells were excluded from analysis. The raw phenotype measurements (percent of cells with a high ratio of blue-to-green fluorescence) were normalized by

dividing each value by the mean of media-only control wells from the corresponding plate. The EC_{50} values were estimated using a drFitSpline function from the grofit R package. (log2(x+1) transformed values were used for spline fitting). Note that for some drugs the max effect might not have been reached at the maximum concentration tested, which might result in underestimation of the EC_{50} values).

Chemical class and target assignment

The information about chemical classes and candidate targets was downloaded from DrugBank database (Law et al., 2014). The enrichment of chemical classes and targets in active versus non-active compound groups was calculated using Fisher's test (R studio). Only the primary target was selected for each drug for the enrichment analysis.

Cross-presentation assay

$1x10^5$ MutuDC were seeded in round bottom 96-well plates and incubated with different concentrations of soluble grade VII OVA (Sigma Aldrich Cat#A7641) or endotoxin-free OVA (Hyglos Cat#300036, Invivogen Cat#vac-pova). Minimal peptide OVA257-264 was used as a control for the capacity of DCs to activate T cells. As indicated, MutuDCs were either incubated with OVA for 45 min, followed by a 3.5 h incubation with small molecules or incubated with OVA and small molecules continuously for 5 h. Next, DCs were washed three times with 0.1% (vol/vol) PBS/BSA, fixed with 0.008% (vol/vol) glutaraldehyde for 3 min at 4°C, washed twice with 0.2 M glycine and twice with the T cell growth media. $1x10^5$ B3Z hybridoma cells were added per well. After 16 h, the cells were lysed in a buffer containing 9 mM $MgCl_2$, 0.125% NP40 (Nonidet® P40 substitute, Santa Cruz Cat#sc-29102) 1.7 mM chlorophenol red-β-D-galactopyranoside (CPRG, Roche Cat#10884308001). CPRG conversion by β-galactosidase was measured by optical density at 590 nm.

OTI and OTII activation assays

$1x10^4$ DCs per well were seeded in round bottom 96-well plates and incubated for 5 h with different concentrations of grade VII OVA (Sigma Aldrich Cat#A7641), endotoxin-free OVA (Hyglos Cat#300036), or control minimal peptides (OVA257-264 and OVA323-339). Where indicated, prazosin was added at 10 μM or Poly(I:C) at 5 μg/ml. After 5 h, DCs were washed three times with PBS containing 0.1% (vol/vol) BSA and co-cultured with $1x10^5$ purified OT-I $CD8^+$ or OT-II $CD4^+$ T cells for 16h. For monitoring T cell activation, T cells were stained for CD69 and CD25 and analyzed by flow cytometry.

Cross-presentation of cell-associated antigens

$1x10^5$ (B3Z assay) or $1x10^4$ (OT-I/II assays) MutuDC were seeded in round bottom 96-well plates with 3T3-OVA cells at various 3T3-OVA:MutuDC ratios (1:2, 1:4, 1:8, 1:16, 1:32). The co-cultures were incubated at 37°C in the presence of prazosin (10 μM) or DMSO (1:1000). After 5 h, the co-cultures were washed, fixed and co-incubated with $1x10^5$ B3Z hybridoma cells or washed and co-cultured for 16 h with $1x10^5$ purified OT-I or OT-II T cells. B3Z and OTI/II T cell activation was monitored as described above.

Cell uptake assay

NIH/3T3 were stained with the PKH-26 membrane dye (Sigma Aldrich, Cat#PKH26-GL) following the manufacturer's instructions. $1x10^5$ MutuDC were plated in 96 round bottom-well plates together with PKH-26^+ 3T3s at different 3T3:MutuDC ratios (1:2, 1:4, 1:8, 1:16, 1:32). The co-cultures were incubated in the presence of prazosin (10 μM) or DMSO for 2 h or 5 h at 37°C, 5% CO2, or left on ice for 5 h. Cells were then stained with anti-CD11c-APC (clone HL3, BD PharMingen Cat#550261) and violet live/dead Dye (ThermoFisher Cat#L34955) and fixed to prevent further uptake.

Percentage of PKH-26^+ MutuDCs ($CD11c^+$ cells) was determined. Phagocytic index was calculated by subtracting the percentage of PKH-26^+ cells in $CD11c^+$ gate obtained at 4°C from the percentage of this subset measured at 37°C after 2 h or 5 h of incubation.

DC activation

To assess DC activation, $1x10^5$ MutuDC were seeded in round bottom 96-well plates and incubated for 5h with endotoxin-free OVA (Hyglos Cat#300036) or grade VII OVA (Sigma Aldrich Cat#A7641), in presence or absence of DMSO or prazosin (10 μM). After 5 h, cells were washed twice with medium, cultured for additional 16 h, and finally stained for CD86.

Live microscopy

MutuDCs were seeded in μ-slide 8 well dishes (Ibidi, Cat#80826) and allowed to adhere overnight. All imaging was performed at 37°C with 5% CO_2. Images were acquired on a VisiTech iSIM swept field confocal super resolution system coupled to a Nikon Ti2 inverted microscope stand equipped with a 100x/1.49 NA SR Apo TIRF objective lens. Fluorophores were excited simultaneously with 488 nm and either 561 nm or 640 nm laser light and imaged with two Hamamatsu ORCA-Flash4.0 V3 CMOS cameras via an image splitter (filter: ZT561rdc from Chroma Technology). The images were analyzed in Fiji (Schindelin et al., 2012) and the panels were assembled in Adobe Photoshop.

Galectin 3-YFP accumulation

MutuDCs stably expressing galectin-3-YFP were imaged for 40 min immediately after addition of 20 μM prazosin. For quantification of galectin-3-YFP recruitment, the videos were manually segmented and spots were identified using the "Analyse particles" function. For each cell, sum of spot areas in all frames was used as a measure of galectin recruitment.

Dextran release assays

MutuDCs were pulsed with 1 mg/ml 3000 MW Tetramethylrhodamine (TRITC)-labeled dextran (Cat#D3307, ThermoFisher Scientific) for 45 min, washed extensively, and incubated with indicated compounds for 1 h. To quantify dextran release into the cytosol, the images were segmented using the watershed algorithm and median fluorescence of the all pixels within each cell was used as a measure of cytosolic fluorescence.

Prazosin-BODIPY accumulation

Imaging was performed immediately after addition of 5 μM prazosin-BODIPY, 10 μg/ml BODIPY, 10 mM NH_4Cl, or indicated combinations. Where indicated, MutuDCs were first pulsed with 10 μg/ml WGA-Alexa Fluor 647 (Cat#W32466, ThremoFisher) for 30 min and washed extensively. For quantification of Prazosin-BODIPY accumulation the images were segmented using the watershed algorithm and the number of spots per cell was quantified using the "Analyse particles" function.

MC38-GFP-OVA tumour growth experiments

WT mice were injected subcutaneously with $2x10^6$ OVA-expressing MC38 cells 100 μL of cold-sterile 1x PBS. When tumours became detectable, the animals were injected three times per week with 0.5 mg/mouse of prazosin i.p. Tumor growth was measured three times a week and volume was calculated as (height × width^2)/2 (where width is the shorter measurement). When tumor size reached 1000 mm^3, the mice were euthanised.

B16-OVA tumour growth experiments

WT or NSG mice were injected subcutaneously with $2.5x10^5$ OVA-expressing B16 cells in 100 μL of cold-sterile PBS. When tumors became visible, usually within a week, mice were randomly assigned to different treatment groups. Injections of prazosin (0.5 mg/mouse, i.p.) and/or anti-PD1 antibody (200 μg/mouse, i.p.) were then performed three times per week, starting the day of tumor appearance. Vehicle (cold water and/or PBS) was injected into control mice. Tumor growth was measured three times a week and volume was calculated as above. When tumor size reached 1000 mm^3, mice were euthanized. To control for toxic effects of prazosin, we performed a pilot experiment in which mice were treated for a period of one month with: 0.5 g prazosin in 1 ml, administered i.p., 3x a week (total of 13 injections, total dose: 7.5 g prazosin per mouse); no adverse effects were observed.

The mean growth rate curves were estimated using loess function in R. The statistical significance analysis was performed in Prism using ANOVA with FDR Benjamini-Hochberg correction.

Whole cell proteomics

MutuDCs were grown in SILAC light or SILAC heavy culture medium, in 15 cm dishes, to 70%–90% confluency. SILAC heavy labeled cells were treated with tamoxifen or prazosin (20 μM) for ~4 h at 37°C; SILAC light labeled cells were treated with DMSO (vehicle) only. Cells were incubated for 3h 45 min at 37°C, and harvested. Cell pellets were lysed in SDS buffer (2.5% (w/V) SDS, 50 mM Tris-HCl, pH = 8.0), and incubated at 90°C for 10 min. To shear genomic DNA, lysates were passed through a QIAashredder (-QIAGEN). Lysates were then processed for analysis by mass spectrometry as described below. For the repeat experiment, the SILAC labeling of control and treated cells was swapped. Protein concentrations were estimated by BCA assay. Equal amounts of control and treated samples (i.e., SILAC light and heavy, or vice versa) were pooled, and acetone precipitated as described (Itzhak et al., 2016). Samples were subjected to tryptic digest using the FASP method (Wiśniewski et al., 2009). Peptides were fractionated into six fractions using strong cation exchange (Kulak et al., 2014) (SCX), prior to mass spectrometric analysis.

Proteomic analysis of cytosol

MutuDCs were cultured in SILAC light or SILAC heavy growth medium, in 10 cm dishes, to 70%–90% confluency. SILAC light cells were treated with tamoxifen, prazosin (both at 10 μM), or vehicle (DMSO) for ~4 h at 37°C; SILAC heavy labeled cells were left untreated, and served as internal reference. Treatments were performed in quadruplicate (two pairs of replicates prepared on two different days). Cells were harvested and resuspended in STE buffer (250 mM sucrose, 0.5 mM MgCl2, 0.2 mM EGTA, 25 mM Tris-HCl, pH = 7.5 at 4°C). Aliquots of SILAC heavy labeled cells were mixed with proportional aliquots of the tamoxifen-, prazosin- or DMSO-treated SILAC light cells. Cells were lysed mechanically in a Dounce homogenizer (tight pestle, 40 strokes, on ice). Lysates were centrifuged at 2,000 x g for 10 min at 4°C, to pellet cell debris und nuclei. Post nuclear supernatants were centrifuged at 135,000 x g for 45 min at 4°C to pellet organelles and microsomes. Supernatants were the cytosolic fraction. Protein concentrations were estimated by BCA assay; aliquots were acetone precipitated and subjected to in-solution digest and stage-tip peptide cleanup as previously described (Itzhak et al., 2016), prior to mass spectrometric analysis.

Dynamic organellar maps

Organellar maps were prepared essentially as described (Itzhak et al., 2016), with minor modifications to the protocol. Briefly, MutuDCs were cultured in SILAC light or SILAC heavy growth medium, in 15 cm dishes, to 70%–90% confluency. SILAC light cells were treated with tamoxifen or prazosin (10 μM), or vehicle (DMSO), for 4 h; SILAC heavy labeled cells were treated with vehicle (DMSO), and served as reference. Two dishes were used for each treatment (SILAC light cells), and four dishes to generate the SILAC heavy reference. Unlike in Itzhak et al. (2016), the same reference was used for treated and control maps. Cells were harvested (with the drugs or DMSO added to the PBS (-) cell detachment buffer), chilled on ice, lysed mechanically in STE buffer (250 mM sucrose, 0.5 mM MgCl2, 0.2 mM EGTA, 25 mM Tris-HCl, pH = 7.5 at 4°C), with a Dounce homogenizer, and centrifuged at 1,000 x g for 10 min to pellet nuclei and cell debris. Post-nuclear supernatants of SILAC light labeled cells were then subjected to a series of differential centrifugation steps (4,000 x g for 10 min; 10,000 x g for 15 min; 20,000 x g for 20 min; 40,000 x g for 20 min; 80,000 x g for 30 min). Post nuclear supernatant from SILAC heavy cells was centrifuged once at 80,000 x g for 30 min to obtain the reference fraction. All pellets were resuspended in SDS buffer (2.5% (w/V) SDS, 50 mM Tris-HCl, pH = 8.0), and heated to 90°C for 3 min. Protein concentrations were estimated by BCA assay. Equal amounts of SILAC heavy reference fraction were mixed with each SILAC light subfraction, acetone precipitated and subjected to in-solution digest and stage-tip peptide cleanup as described (Itzhak et al., 2019), prior to mass spectrometric analysis.

Fractionations were prepared in duplicate, on two different days (six maps total – two controls, two from cells treated with tamoxifen, and two from cells treated with prazosin).

Mass spectrometry and data processing

Mass spectrometric analysis was performed as described (Itzhak et al., 2016), using a Thermo EASY-nLC 1000 HPLC coupled to a Q Exactive HF Hybrid Quadrupole-Orbitrap (Thermo Fisher Scientific, Germany). HPLC gradient lengths varied for the different experiments. For analysis of whole proteomes, each of the SCX peptide fractions was analyzed with a 240 min gradient (24 h per sample in total). For the analysis of cytosol and fractions from the organellar maps, each sample was analyzed with a single 150 min gradient. Raw files were processed with MaxQuant software Version 1.6 (Cox and Mann, 2008), using the murine reference proteome (Swiss-Prot canonical and isoform data) database downloaded from UniProt (https://www.uniprot.org:443/).

Bioinformatic analysis of the proteomic data

Protein groups identified through MaxQuant analysis were filtered to remove reverse hits, proteins identified with modified peptides only, as well as common contaminants. Further processing depended on the individual experiment:

Copy number estimates of proteins expressed in MutuDC

To estimate absolute protein abundance in MutuDCs, the SILAC datasets used for full proteome analysis of drug-treated cells were used (see below). Each of the four dataset combined control cells and drug treated cells. From each dataset, the protein intensities from the control cells were selected, to obtain four replicate full proteomes. Intensities within each replicate were summed, and all replicates were linearly normalized to the same summed intensity. Next, only proteins detected in at least two replicates were retained (7427 in total). Copy number estimates were calculated using the Proteomic Ruler (Wiśniewski et al., 2014), as implemented in Perseus software (V1.5) (Tyanova et al., 2016), and described in Itzhak et al. (2019). Protein intensities were scaled to molecular mass.

Drug-induced changes in whole cell proteomes

For analysis of drug-induced changes in whole cell proteomes, only proteins with at least three SILAC quantification events in each of the four experiments (2 x control versus tamoxifen, 2 x control versus prazosin) were retained (5848 proteins). SILAC ratios were linearly normalized to a column median of 1 in each experiment, logarithmised, and analyzed with the 'Significance A' tool in Perseus software (Tyanova et al., 2016). Proteins that changed significantly in both replicate experiments with one drug (FDR = 0.05 within each replicate, Benjamini-Hochberg correction), with a consistent direction of change, were considered as hits for this drug. Proteins that changed significantly across both replicates and both drug treatments, with a consistent direction of change in all four measurements, were considered as hits common to both drugs.

Drug-induced changes in cytosol

For analysis of drug-induced changes in cytosol, only proteins with at least three SILAC quantification events in each of the four replicates were retained (2129 proteins). SILAC ratios were linearly normalized to a column median of 1 in each experiment, and logarithmised. For each protein, the average ratio SILAC light/SILAC heavy from the four replicates was calculated for each condition, and average control (DMSO) ratios were then subtracted from average treatment (tamoxifen or prazosin) ratios. Thus, for every protein, the average change in cytosolic levels caused by either tamoxifen or prazosin relative to DMSO was obtained.

The log ratios from the whole cell proteome and cytosol analyses were plotted against each other for each treatment (including only proteins detected in both). To compare the distribution of lysosomal proteins with the distribution of all detected proteins, a Kolmogorov–Smirnov test was performed.

Organellar maps

Generation of organellar maps and outlier testing followed the principles described in detail (Itzhak et al., 2019; 2016), with some modifications to accommodate a comparison across three conditions. Only proteins with high quality SILAC ratios in all 30 subfractions, i.e., across all six maps, were retained (1857 proteins). (High quality SILAC ratios are those calculated from three or more quantification events. In addition, ratios calculated from only two quantification events are also included in the high quality set if the corresponding MaxQuant ratio variability was below 30%). Each map consisted of a set of five SILAC ratios for each protein, mirroring its distribution across the differential centrifugation fractions. SILAC ratios were inverted, and divided by the sum of all five ratios across the map. This yielded for each protein a 'per map' normalized profile (summing to 1). For the MutuDC control map shown in 2C and Figures 3B, all proteins passing the high quality filter in both replicates were included (2121 proteins). To visualize the map the prcomp function in R was used, with the following parameters: (center = TRUE, scale. = TRUE). Organellar marker proteins were initially chosen from our previously published set, and augmented as described (Itzhak et al., 2016).

Subcellular localization predictions in MutuDC

Organellar maps from the two control map replicates (0-1 normalized, Data S2) were annotated with 559 markers for 12 organellar compartments, by cross-matching our previously derived set of human marker proteins (Itzhak et al., 2016). Support vector machines (implemented in Perseus software, V1.6) (Tyanova et al., 2016) were trained to predict organellar association as described (Itzhak et al., 2016), with an overall recall of 93% and a median F1 score of 0.88 across all compartments (Data S2).

Drug induced protein movements

To identify proteins that moved significantly and robustly, our previously reported MR (movement and reproducibility) (Itzhak et al., 2016) analysis was applied, with minor modifications. Unlike in our previous study, here only one reference fraction was used to generate the control and treatment maps. This reference came from cells treated with DMSO only. A different normalization was therefore required, to allow the outlier test to detect changes in membrane association as well as organellar localization shifts. SILAC ratios were first normalized within each fraction to a column median of 1. Next, for each protein, SILAC ratios were inverted, and weighted with fraction yields (determined by BCA assay) (Itzhak et al., 2016). Within each map all data were then summed. This reflected overall amount of protein detected in each map (prep yield). The smallest prep yield was set to one, and correction factors for the other five maps were calculated relative to this value. All data within a map were then globally normalized through division by the prep yield correction factor. The result were six maps in which the sum of all data points is equal. Next, for each protein the ten data points from the two tamoxifen replicates and the ten corresponding data points from control replicates were divided by the sum of all of these ratios. The same was repeated for the ten data points from the two prazosin replicates, using the same ten control data points. This procedure results in an additional "within treatment" normalization of the maps. Next, for each protein, the treatment profiles were subtracted from the corresponding control profiles, to yield 'delta' profiles. For every protein, four delta profiles, with five data points each (two sets from tamoxifen and two sets from prazosin treatment) were obtained. Delta profiles from treatment replicates were combined into one profile (ten data points) and analyzed with the multivariate outlier test in Perseus software (Perseus 1.6, 101 iterations, quantile = n*0.75) (Itzhak et al., 2016). Movement (M) scores were calculated as the negative log(10) of the FDR corrected p values (Benjamini-Hochberg method). For example, an M score of four identifies significantly moving proteins with an FDR of 0.01%. The reproducibility (R) score was calculated as the Pearson correlation of the two five-data point delta profiles within treatment replicates. A significance cut-off corresponding to a p value of 0.05 (R = 0.8) was chosen. Since the R-score represents an additional filter, orthogonal to the M-score, further multiple hypothesis correction of the p value was not required. Each protein with significant M (> 4) and R (> 0.8) scores was considered as shifting significantly. Thus, for every protein two sets of M and R scores were obtained, reflecting shifts caused by tamoxifen or prazosin treatment. Each treatment produced a partially overlapping list of shifting proteins.

QUANTIFICATION AND STATISTICAL ANALYSIS

Details of the statistical analysis are provided in Figure Legends and in STAR Methods. Plots were generated using GraphPad Prism version 8.4.2 for Mac (GraphPad Software, La Jolla California USA) or R (R Core Team, R: A Language and Environment for Statistical Computing (Version 3.5.0, R Foundation for Statistical Computing, Vienna, 2018; https://www.R-project.org/) and Tidyverse (Wickham et al., 2019).

ADDITIONAL RESOURCES

A web resource to mine proteomics data associated with the study is available at http://dc-biology.mrc-lmb.cam.ac.uk

Cell Reports, Volume *32*

Supplemental Information

Small Molecule Enhancers of Endosome-to-Cytosol Import Augment Anti-tumor Immunity

Patrycja Kozik, Marine Gros, Daniel N. Itzhak, Leonel Joannas, Sandrine Heurtebise-Chrétien, Patrycja A. Krawczyk, Pablo Rodríguez-Silvestre, Andrés Alloatti, Joao Gamelas Magalhaes, Elaine Del Nery, Georg H.H. Borner, and Sebastian Amigorena

Figure S1

ERAD inhibitors

Importazole

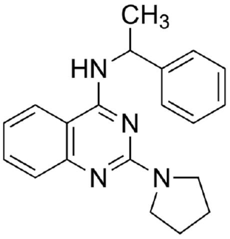

DbeQ

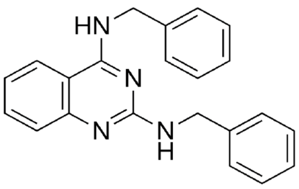

FDA library

Active compounds

Prazosin

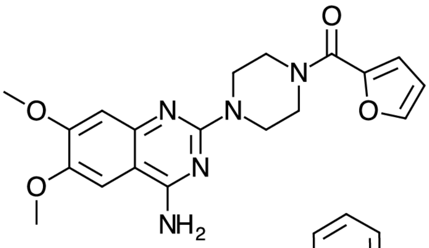

Doxazosin

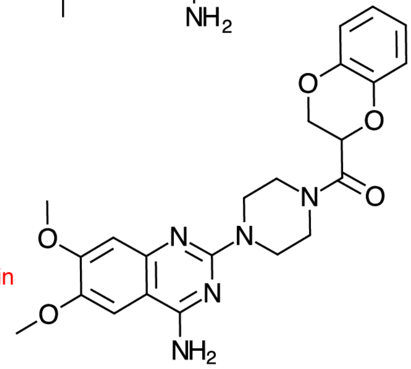

Gefitinib

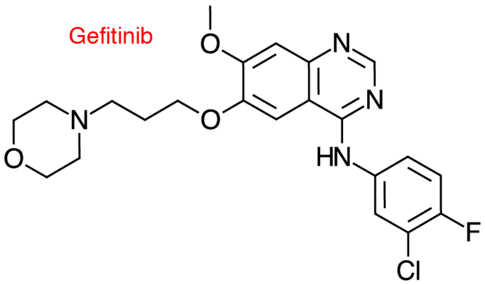

Non-active compounds

Terazosin

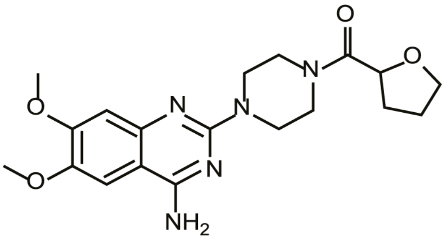

Lapatinib

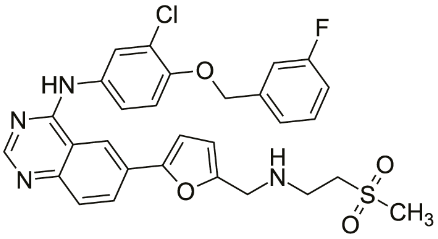

Figure S1. Chemical structures of the ERAD inhibitors and of selected active and non-active quinazolinamine tested in this study.

Related to Figure 1

Figure S2

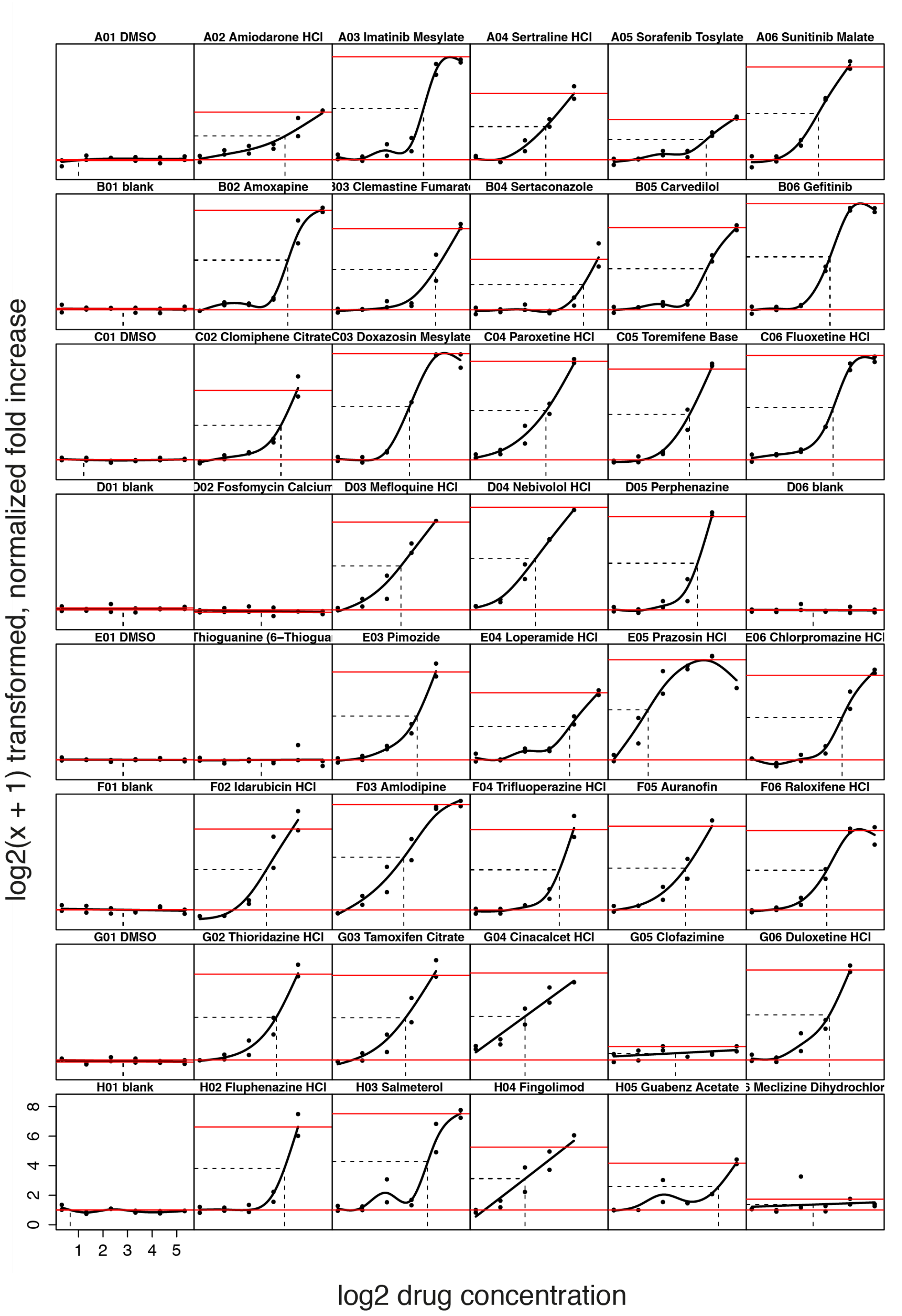

Figure S2. Summary of the EC50 plots from the secondary screen.

Related to Figure 1

39 compounds were analysed using the β-lactamase assay: 37 top ranked compounds from the primary screen and two compounds with no phenotype were included (fosfomycin calcium and thioguanine). No treatment (blank) and vehicle (DMSO) controls were included on each plate. The screen was performed with five doses for each of the drugs (1.25 - 40 μM). Wells with fewer than 500 cells were excluded from the analysis. The proportion of the cells with efficient β-lactamase translocation was determined and these raw phenotype

measurements were normalised by dividing each value by the mean of the "DMSO" control wells from the corresponding plate. drFitSpline function from the grofit R package was used to estimate the yEC50 (50% of the max effect) values. The fold-increase and concentration values were log2(x+1) transformed for spline fitting. The concentration values were log2 transformed. The red lines indicated max and min import efficiency, the horizontal dotted line indicates the yEC50 value and the vertical line the corresponding compound concentration.

Figure S3

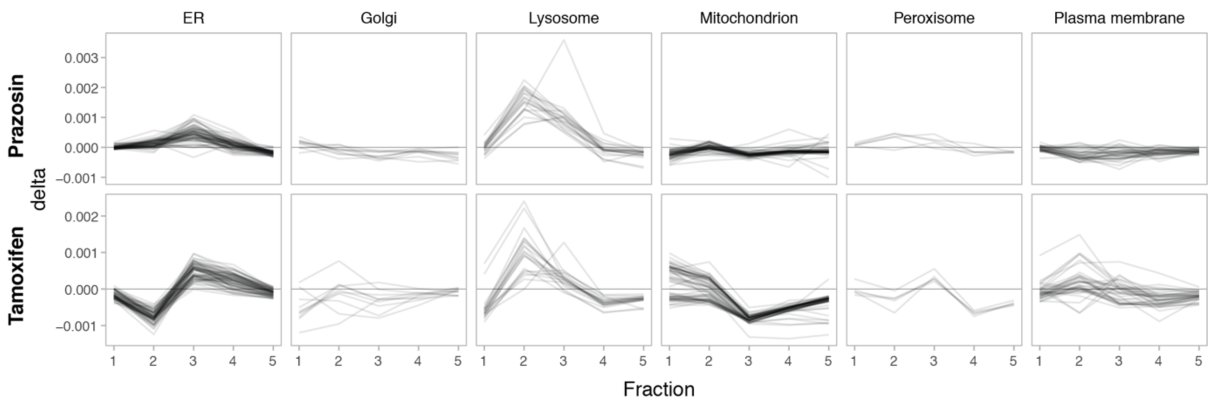

Figure S3. 'Movement' data for proteins from selected organelles used for 'MR' analysis.

Related to Figure 3.

Prazosin- and tamoxifen-induced shifts in protein subcellular localization detected using 'MR' plot analysis. Each line represents the normalised shift for one protein (average from two independent experiments). All proteins assigned to the five organelles represented in the panel are shown.

Figure S4

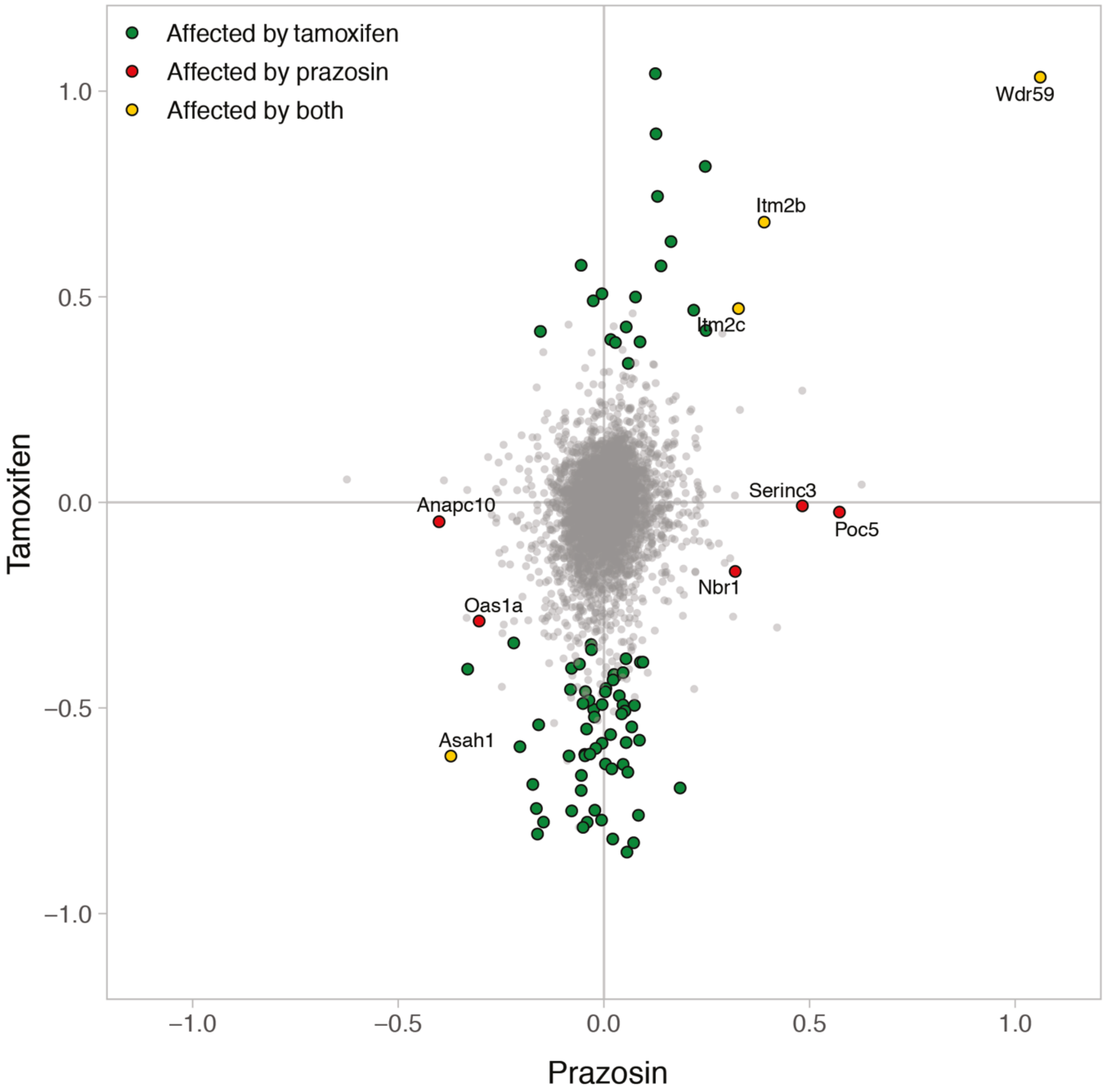

Figure S4. Whole cell protein expression levels in MutuDCs.

Related to Figure 3

Protein expression levels in MutuDCs from prazosin or tamoxifen treated (4 h) cells relative to untreated controls (log 2 scale). Quantification was achieved by metabolic labelling (SILAC; averages of two replicates are shown). 5848 proteins were quantified across all four experiments. Proteins that changed significantly in abundance are highlighted in colour. Tamoxifen treatment induced more changes in proteome composition than prazosin treatment; overall, the changes induced by either treatment were relatively moderate.

Figure S5

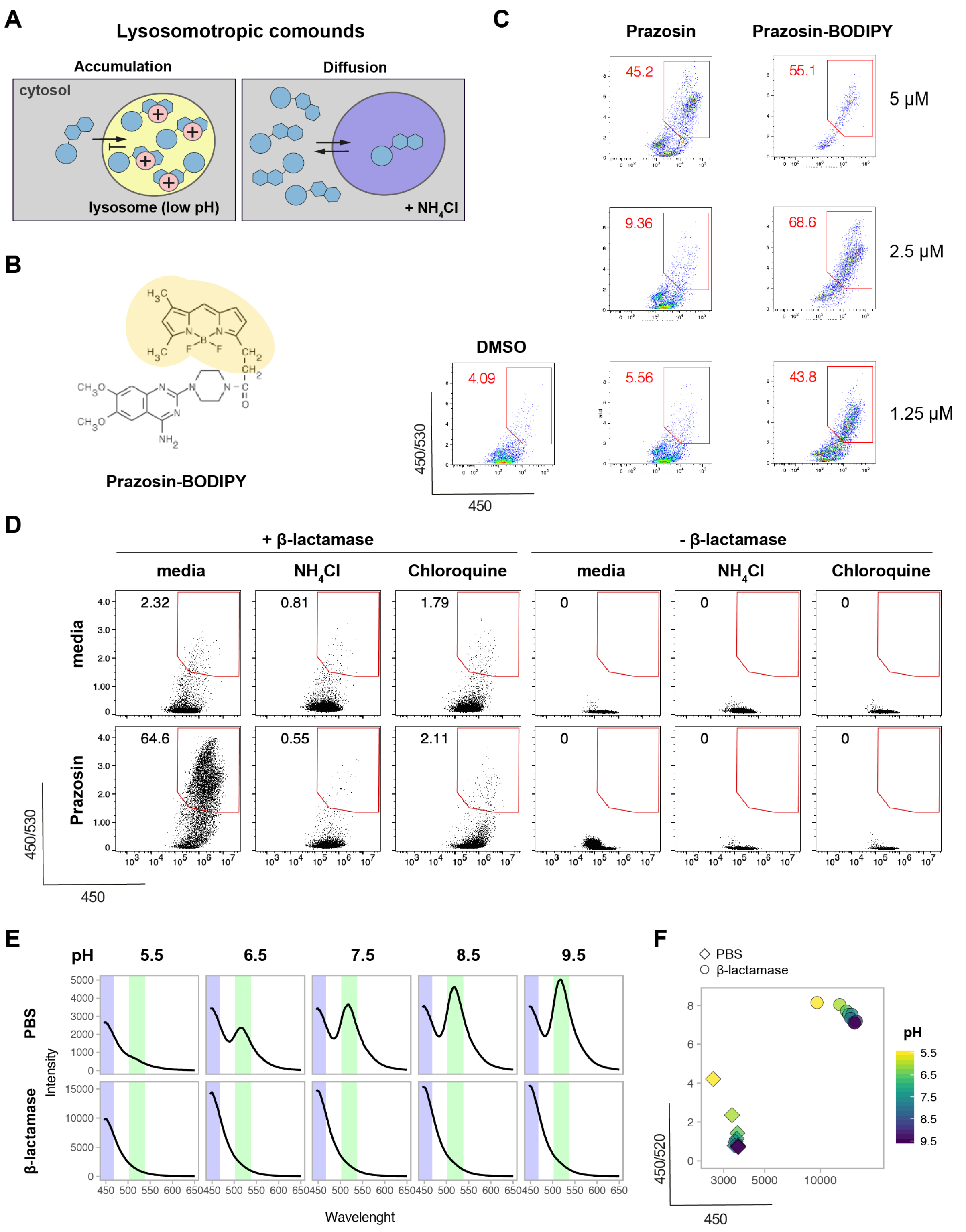

Figure S5. Prazosin activity is linked to accumulation in low pH compartments.

Related to Figure 5

A. Schematic representation of lysosomal trapping. Membrane permeable small molecules diffuse freely across the membrane of acidic compartments. Protonation of weakly basic residues decreases membrane permeability

and leads to accumulation of protonated compounds in the endo-lysosomal lumen. When the pH of intracellular compartments is neutralised, the compounds are no longer protonated and are free to diffuse across the membrane.

B. Comparison of the chemical structures of prazosin and prazosin-BODIPY.

C. Activity of prazosin-BODIPY in the β-lactamase assay. Prazosin modified with BODIPY is active in the antigen import assay.

D. Antigen import assay was performed in the presence of NH_4Cl or chloroquine with or without prazosin. NH_4Cl or chloroquine alone have no effect on antigen import and they both abolish prazosin activity. Cells with the high blue-to-green fluorescence ratio are absent in samples that were not pulsed with β-lactamase.

E and F. To determine whether the β-lactamase assay is sensitive to changes in cytosolic pH, CCF2-FA was incubated in PBS with or without β-lactamase for 90 min (CCF2-FA is a free acid version of the CCF4-AM and it does not require de-esterification by the cytosolic esterases). The fluorescence spectra of cleaved and uncleaved CCF2 were then determined in buffers of different pH using Tecan Spark microplate reader (excitation wavelength: 405 nm). E. Sample spectra for cleaved and uncleaved CCF2. The wavelengths collected during flow cytometry are indicated in blue and green. F. Ratio of blue (emission at 450 nm) to green (emission at 520 nm) fluorescence plotted against fluorescence at 450 nm (similar to the representations for the flow cytometry data). While acidification results in quenching of both CCF4/2 subunits, fluorescein and coumarin, the cleaved and uncleaved dye remain separated.

Figure S6

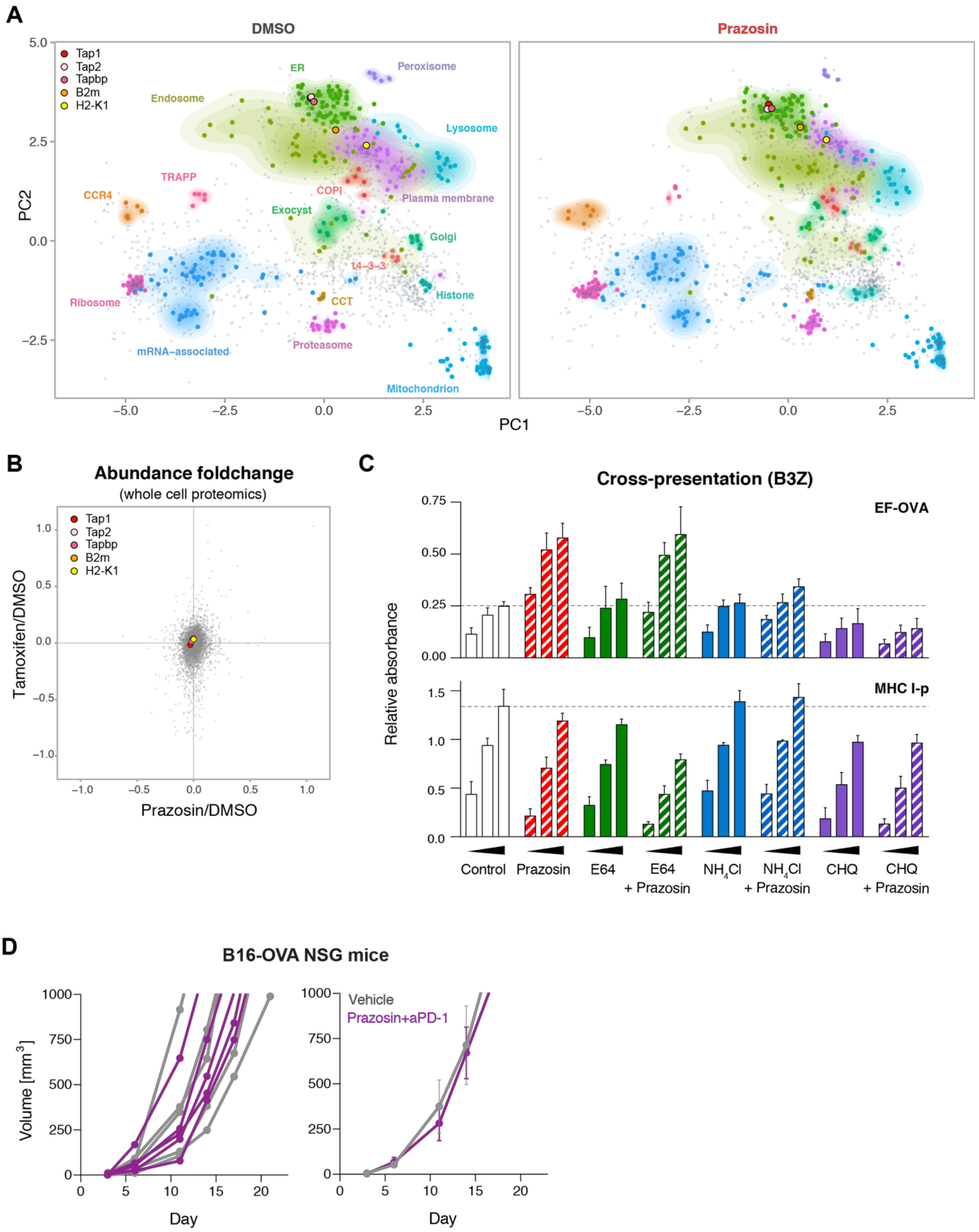

Figure S6. Analysis of the prazosin activity in antigen cross-presentation in vitro and control of tumour growth in vivo.

Related to Figure 6

A. Organellar maps (see Figure 3B) of prazosin- and DMSO-treated cells. Prazosin does not lead to re-localisation of the loading complex or MHC I subunits.

B. Fold increase (log2 scale) of the loading complex components and MHC class I subunits in drug-treated samples (relative to DMSO control) (see Figure S4). Neither prazosin nor tamoxifen lead to a change in abundance of the loading complex components or of the MHC I.
C. MutuDCs were incubated with sOVA EF or MHC I peptide for 5 h, in the presence of indicated compounds. Cross-presentation efficiency was monitored using the B3Z assay. Neutralisation of endolysosomal compartments or inhibition of cathepsins are not sufficient for cross-presentation enhancement in MutuDCs. NH_4Cl and chloroquine abolish the prazosin effect on cross-presentation, similar to the phenotypes observed in antigen import assays.
D. Tumour growth curves for NSG mice injected s.c. with the B16-OVA tumour cells. From the day when tumours became detectable, mice were injected i.p. three times per week with a combination of 0.5 mg prazosin and 150 µg anti-PD-1.
